# β2 spectrin-mediated differentiation repressed the properties of liver cancer stem cells through β-catenin

**DOI:** 10.1038/s41419-018-0456-6

**Published:** 2018-03-19

**Authors:** Yuhua Chen, Lingling Meng, Haitao Shang, Qian Dou, Zhiwen Lu, Liping Liu, Zhijun Wang, Xingxing He, Yuhu Song

**Affiliations:** 1 Division of Gastroenterology, Union Hospital, Tongji Medical College Huazhong University of Science and Technology, Wuhan, 430022 China; 2 Institute of Liver Diseases, Tongji Hospital, Tongji Medical College Huazhong University of Science and Technology, Wuhan, 430030 China

## Abstract

βII-Spectrin (β2SP), a Smad3/4 adaptor protein during transforming growth factor (TGF) β/Smad signal pathway, plays a critical role in suppressing hepatocarcinogenesis. Dedifferentiation is a distinctive feature of cancer progression. Therefore, we investigated whether the disruption of β2SP contributed to tumorigenesis of hepatocellular carcinoma (HCC) through the dedifferentiation. Down-regulation of β2SP in hepatocytes was observed in cirrhotic liver and HCC. The level of β2SP expression was closely associated with the differentiation status of hepatocytes in rat model of hepatocarcinogenesis and clinical specimens. Transgenic expression of β2SP in HCC cells promoted the differentiation of HCC cells and suppressed the growth of HCC cells in vitro. Efficient transduction of β2SP into liver CSCs resulted in a reduction in colony formation ability, spheroid formation capacity, invasive activity, chemo-resistance properties, tumorigenicity in vivo. In addition, β2 spectrin exerted its effect through β catenin in liver CSCs. In conclusion, β2 spectrin repressed the properties of liver CSCs through inducing differentiation; thus, strategies to restore its levels and activities would be a novel strategy for HCC prevention and differentiation therapy

## Introduction

Hepatocellular carcinoma (HCC), the most common solid tumors, is the second most common cause of cancer-related deaths worldwide with a poor survival rate^1^. The etiology of HCC includes hepatitis virus, chronic alcohol consumption, nonalcoholic steatohepatitis, exposure of hepato-toxins, and etc. Despite advances in the detection and treatment of HCC, most patients with HCC have an extremely poor prognosis because they are diagnosed at an advanced stage. Hepatic tumor progression is defined by progressive deterioration in cell differentiation, accumulation of genomic aberrations, an extinction of tissue-specific gene expression, acceleration of cell proliferation, increased invasiveness, early metastasis, and high-grade malignancy^2,3^. Of critical steps of liver tumorigenesis, hepatocyte dedifferentiation is a key cellular event^4^. HCC progression from a well differentiated to a less differentiated form is accompanied by a dramatic alteration in the morphological and genetic properties of hepatocytes^2^. Previous studies demonstrated that differentiation therapy represented a promising therapeutic method through inducing the differentiation of hepatoma cells into mature hepatocytes in animal model of HCC^5–7^. Accumulating evidences in animal models of solid tumors suggest that oncogenic mutations and/or epigenetic aberrations in a more differentiated cell generate continuously proliferating cells that no longer enter a post-mitotic differentiated state, thereby creating a pool of self-renewing cells in which further mutations can accumulate^8,9^. A pool of self-renewing cells within the tumor mass called cancer stem cells (CSCs) or tumor-initiating cells (T-ICs) have the ability to self-renew, differentiate into defined progenies and, most importantly, initiate and sustain tumor growth^9–11^. Liver T-ICs play a major role not only in initiating and sustaining primary tumors but also in facilitating metastasis to distant organs. The aggressive phenotypic traits of primary liver cancers with respect to self-renewal, tumorigenicity, invasiveness, and chemoresistance are assumed to be dependent on CSCs or T-ICs^9–11^. Thus, an effective strategy for cancer treatment should be developed through inducing CSCs differentiation by key transcription^5,6^.

Transforming growth factor-β (TGF-β) signaling pathway plays a critical role in stem cell renewal and differentiation^12^. Deregulation of TGFβ signaling potentially contributes to impaired differentiation and allows for the development of cancers, linking the differentiation of stem cells with suppression of carcinogenesis. The adaptor protein, βII-Spectrin (β2SP), plays an essential role in translocating the Smad3/Smad4 complex into the nucleus, and then drives TGFβ-mediated tumor suppression^13–19^. Thus, the disruption of TGFβ signaling by loss of β2SP is critical to the development of gastrointestinal cancers^13–19^. Interestingly, previous study demonstrated that loss of β2SP was associated with activation of liver progenitor cells secondary to delayed mitogenesis^20^. Zhi et al suggest that knockdown of β2SP expression promoted acquisition of stem cell-like feature in HCC cells, and ultimately contributed to malignant tumor progression^17^. Thus, we hypothesized that loss of β2SP resulted in HCC through disruption of a normal pattern of cellular differentiation. However, the role of β2SP in the differentiation of HCC has not been reported, so far. In this study, we clarified, for the first time, that β2SP expression correlated with the differentiation of hepatocytes, and β2SP-mediated differentiation suppressed the growth of HCC cells in vitro. Moreover, we showed that differentiation induced by β2SP dramatically suppressed the features of liver CSCs. Strategies to restore its levels and activities could be a novel strategy for HCC prevention and differentiation therapy.

## Materials and methods

### See Supplementary Methods for detailed experimental methods

#### Cell lines, tumor specimens and animal

HepG2, SMCC7721, PLC/PRF/5, Huh7 cells were cultured in Dulbecco’s modified essential medium (DMEM) supplemented with 10% fetal bovine serum (FBS) in humidified air containing 5% CO_2_ at 37 °C. SUN-398 cells were cultured in Roswell Park Memorial Institute 1640 (RPMI-1640) supplemented with 10% FBS. Human liver cancer specimens and normal liver specimens were obtained from Department of Hepatobiliary Surgery, Union Hospital, Tongji Medical College (Wuhan, China) with informed consent according to the Institutional Review Board approval. The rat model of liver fibrosis and HCC was induced by intraperitoneal injection of diethylinitrosamine (DEN).

### The effect of β2SP on the growth of HCC cells in vitro

Human β2SP was cloned by PCR from the reverse transcribed complementary DNA of HepG2 cell RNA. HCC cells were transfected with the plasmids carrying β spectrin using Lipofectamine 2000. The effects of β2SP on the growth of HCC cells in vitro were assessed by the expression of cell cycle regulatory proteins, cell cycle distribution using flow cytometry, CCK8 cell proliferation assay^21–24^. Real-time reverse transcription PCR (RT–PCR) was performed to determine the transcripts of liver CSCs markers and liver-specific genes.

### The isolation of liver CSCs and functional assays

CD133^+^ or EpCAM^+^ populations were isolated from HCC cells using CD133/EpCAM MicroBeads kit (Miltenyi Biotec, Bergisch Gladbach, Germany). The purity of sorted cells was detected by flow cytometry. Sorted cells were transfected with the plasmids carrying β spectrin using Lipofectamine 2000, and then in vitro tumorigenic abilities were assessed by spheroid formation, colony-formation assays, chemo-resistant assays, and invasion assay. In addition, *In vivo* the tumorigenicity of liver CSCs was investigated in xenograft models of NOD-SCID mice.

## Results

### Expression of β 2 spectrin was reduced during hepatocarcinogenesis

**Fig. 1:**
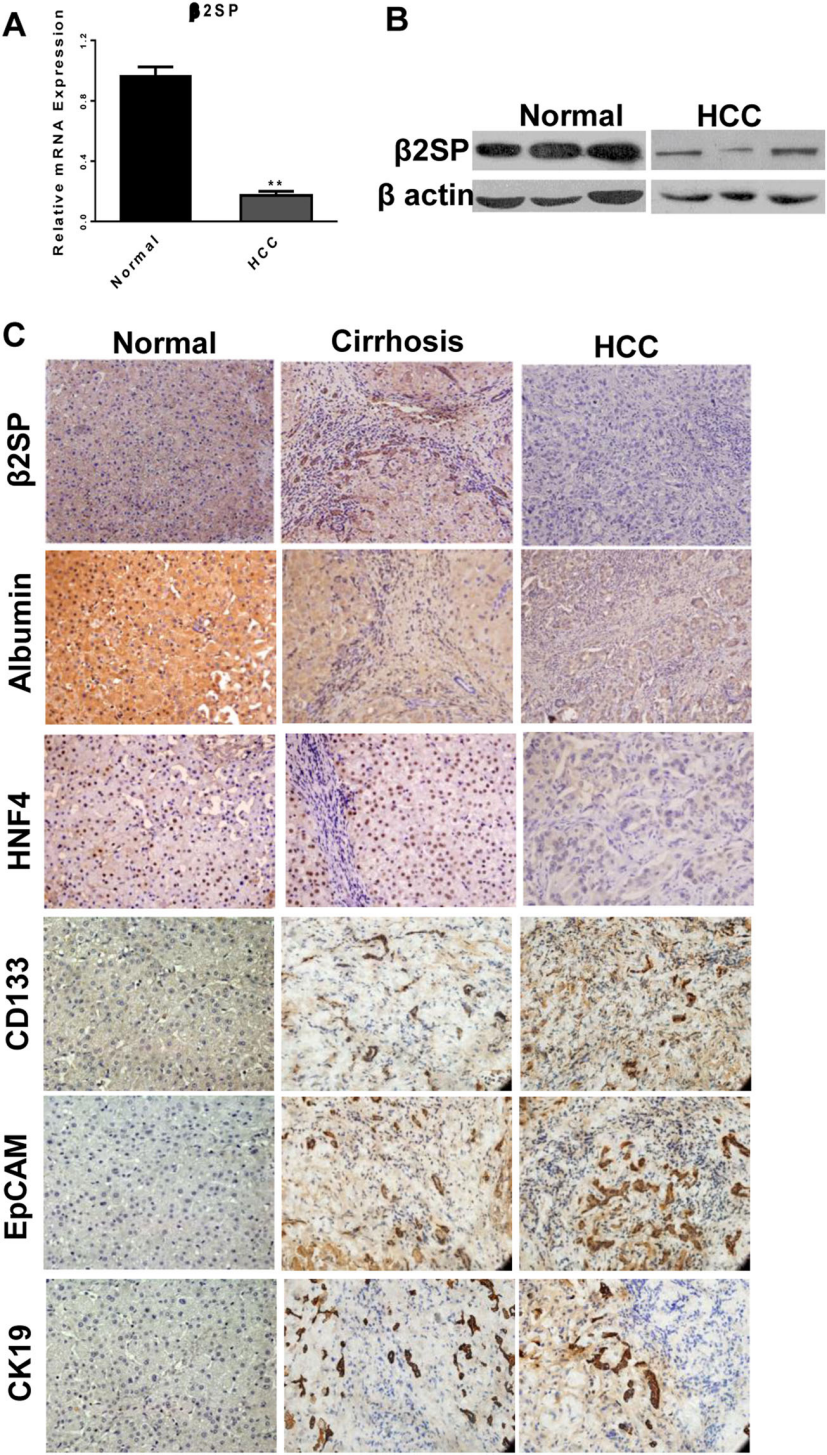
The expression of β2 spectrin correlated with hepatocyte differentiation in the patients with liver cirrhosis and HCC. **a** Quantification of β2 spectrin mRNA transcript in whole liver homogenates from normal and HCC patients by real-time RT–PCR. Normal liver tissues refers to normal tissues obtained from surgical resection due to hepatic hemangioma; **b** Quantification of β 2 spectrin protein in whole liver homogenates from normal and HCC patients by Western blot; **c** Immunohistochemical staining determined the expressions of β2SP, liver-specific genes (albumin, HNF4α), and liver cancer stem cell markers (CD133, EpCAM, CK19) in the patients with normal, liver cirrhosis and HCC. Magnification, ×200. All the data were means ± SEM of three independent experiments (**P < 0.01, *P < 0.05)

**Fig. 2:**
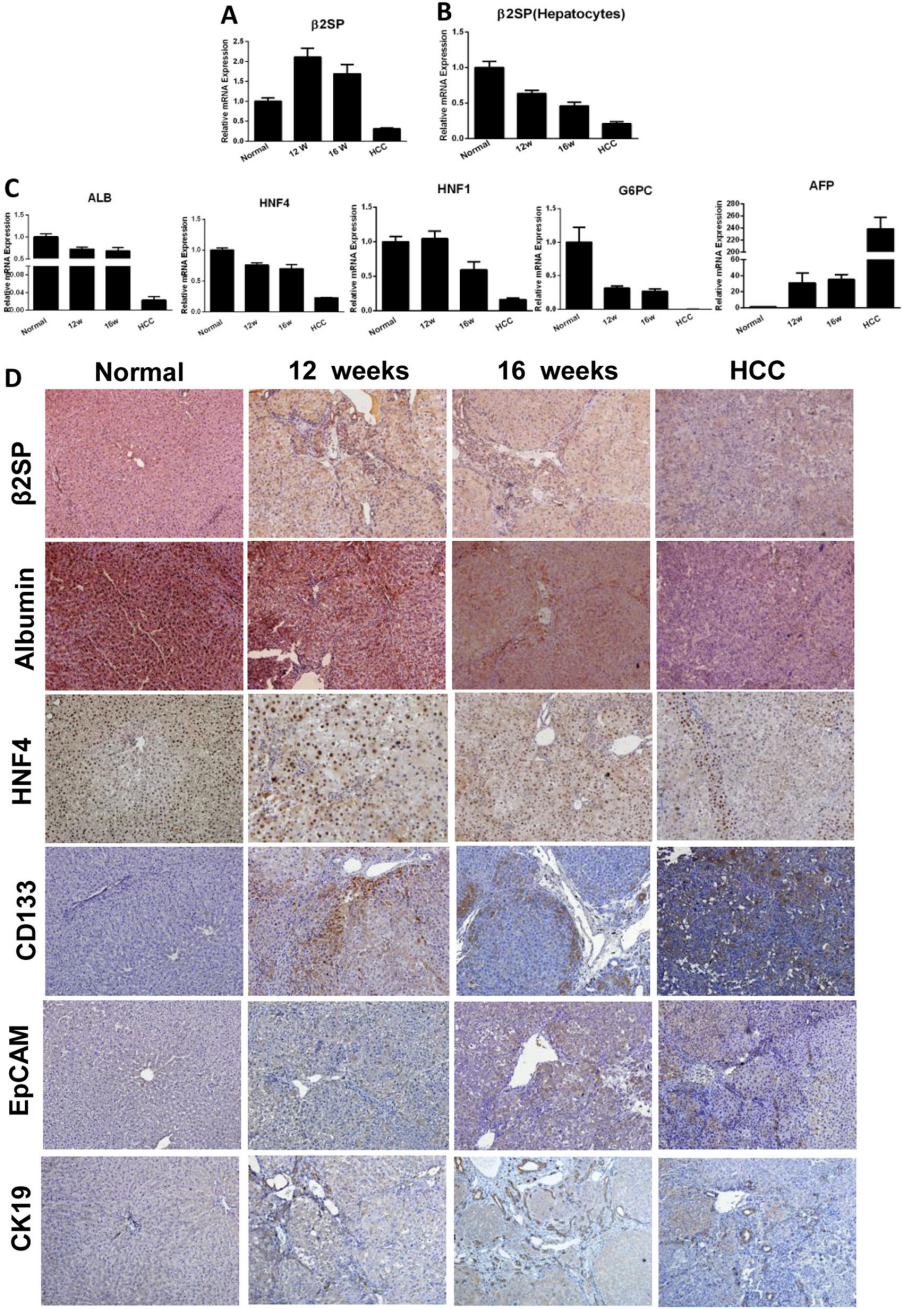
β 2 spectrin expression correlated with hepatocyte differentiation in DEN-induced rat model of liver fibrosis and HCC. **a** Quantification of β 2 spectrin mRNA transcript in whole liver homogenates from control and DEN-treated rats by real-time RT–PCR. **b** Real-time RT–PCR determined β 2 spectrin mRNA transcript in hepatocytes isolated from control or DEN-treated rat. The results showed that the transcripts of β 2 spectrin were reduced in hepatocytes during hepatocarcinogensis. **c** Real-time RT–PCR quantification of liver-specific genes (albumin, HNF4α, HNF1α, G6PC) and α fetoprotein in DEN-treated rats. **d** Immunohistochemical staining determined the expressions of β2SP, liver-specific genes (albumin, HNF4α), and liver cancer stem cell markers (CD133, EpCAM, CK19) in DEN-treated rats. Magnification, ×200. All the data were means ± SEM of three independent experiments (**P < 0.01, *P < 0.05)

Given the prominent role of β2SP in the tumor-suppressive activity of TGFβ signaling, β2SP expression was determined in human samples and diethylnitrosamine (DEN)-treated Wistar rats during hepatocarcinogenesis. First, β2SP expression in liver tissues was examined by real-time RT–PCR, western blot and immunohistochemical staining in humans with normal, liver cirrhosis, and HCC. As shown in Fig. 1a, b, downregulation of β2SP expression was detected in HCC tissues compared with normal tissues. Immunohistochemical staining (Fig. 1c) and immunofluorescence (Fig. S1) showed enhanced β2SP expression in activated HSCs of portal area and decreased β2SP expression in hepatocytes of regenerative nodule, which was consistent with our previous study in CCl_4_-treated fibrotic mice^25^. Second, β2SP expression was evaluated in liver samples from DEN-administrated rats. Similar results (Fig. 2a, d, Fig. S3) were also demonstrated in DEN-induced cirrhotic and HCC liver tissues. Importantly, hepatocytes isolated from normal or cirrhotic liver were subjected to RT–PCR analysis, and qRT–PCR results (Fig. 2b) also confirmed that β2SP expression decreased gradually in hepatocytes during hepatocarcinogenesis. All these indicated β 2 spectrin expression was progressively reduced in the hepatocytes during hepatocarcinogenesis.

### β 2 spectrin expression correlated with hepatocyte differentiation

To explore the relevance of β2SP expression with the status of hepatocyte differentiation, the expression of liver-specific genes and liver CSCs markers (stemness-associated genes) was determined in human samples and DEN-administrated rats. Immunochemical staining (Figs. 1c and 2d) showed that reduction of β2SP expression was in concomitance with the downregulation of liver-specific genes (albumin, HNF4α) in human or rat liver tissues during hepatocarcinogenesis. Simultaneously, expression of liver CSC markers (CD133, EpCAM, CK19) increased progressively (Figs. 1c and 2d). In addition, qRT–PCR confirmed β2SP expression correlated with the expression liver-specific genes in DEN-treated rat (Fig. 2c). All these indicated that β2SP expression correlated with the differentiation status of hepatocytes during hepatocarcinogenesis.

### β2 spectrin suppressed the growth of HCC cells in vitro

**Fig. 3:**
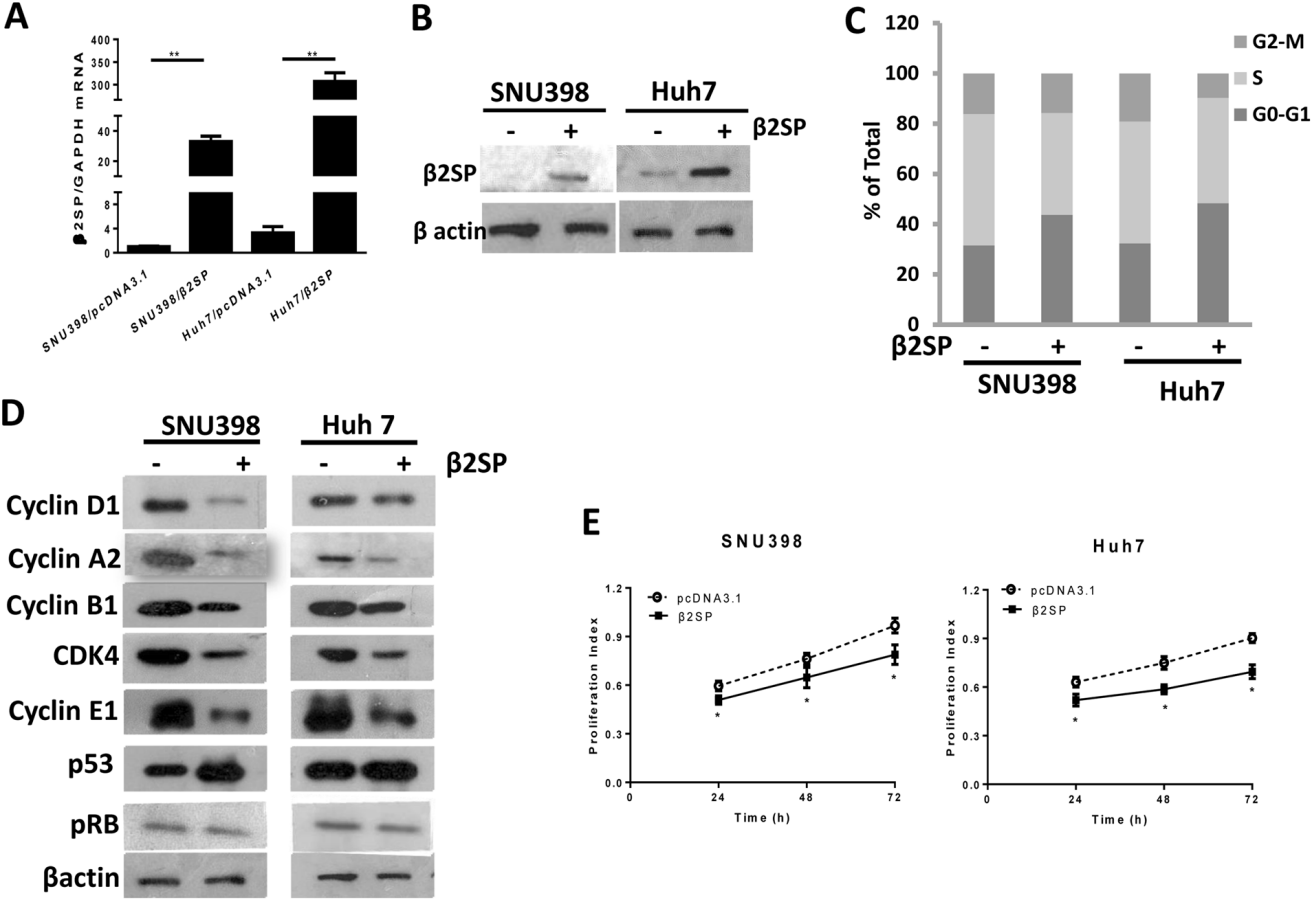
β2SP suppressed the growth of HCC cells (SUN398 and Huh7 cells) in vitro. The plasmids expressing β2SP were transfected into SUN398 and Huh7 cells. 48 h later, the cells were collected for further studies. **a** Quantitative RT–PCR determined β2SP mRNA in HCC cells transfected with the vector expressing β2SP cDNA or the control; **b** Western blot analysis determined β2SP protein in HCC cells transfected with the vector expressing β2SP cDNA or the control; **c** the percentages of cell distribution in each phase were determined in transfected HCC cells by flow cytometry. Percentages of cell distribution in each phase were shown on the histogram. **d** the expression patterns of cell cycle regulatory proteins were analyzed by western blot **e** the proliferation of HCC cells was evaluated by Cell Counting Kit-8 (CCK8). All the data were means ± SEM of three independent experiments (**P < 0.01, *P < 0.05)

To investigate the tumor-suppressive role of β2SP, the plasmid carrying human β2SP cDNA was transfected into HCC cell lines (SNU-398 and Huh 7 cells), and then functional assays in vitro were performed to assess tumor-suppressive role of β2SP in HCC cells. Firstly, we determined the level of β2SP expression in different HCC cells, and the result of qRT–PCR showed low level of β2SP expression in Huh7 and SNU-398 cells (Fig. S4). Therefore, Huh7 and SNU-398 cells were used in further study. Secondly, we determined β2SP expression in transfected HCC cells, the results (Fig. 3a, b) revealed efficient expression of β2SP in HCC cells after transfection of β2SP. Thirdly, we evaluated suppressive effect of β2SP on the growth of HCC cells *in vitro*. Cell cycle assay using flow cytometry demonstrated cell cycle arrest in HCC cells transfected with β2SP cDNA, which was indicated as significant change in distribution of S phase and G0-G1 phase (Fig. 3c). In addition, overexpression of β2 spectrin markedly suppressed the expression of the proteins responsible for cell cycle checkpoint such as CDK4, cyclin D1, cyclin A2, cyclin B1, and pRb, at the same time, stabilized p53 (Fig. 3d) As show in Fig. 3e, cell proliferation assay showed that proliferative capacity of HCC cells decreased significantly upon the treatment of β2SP. All these revealed the tumor-suppressive role of β 2 spectrin in HCC cells in vitro.

### β2 spectrin promoted the differentiation of hepatoma cells and repressed transcription of liver cancer stem cell markers

**Fig. 4:**
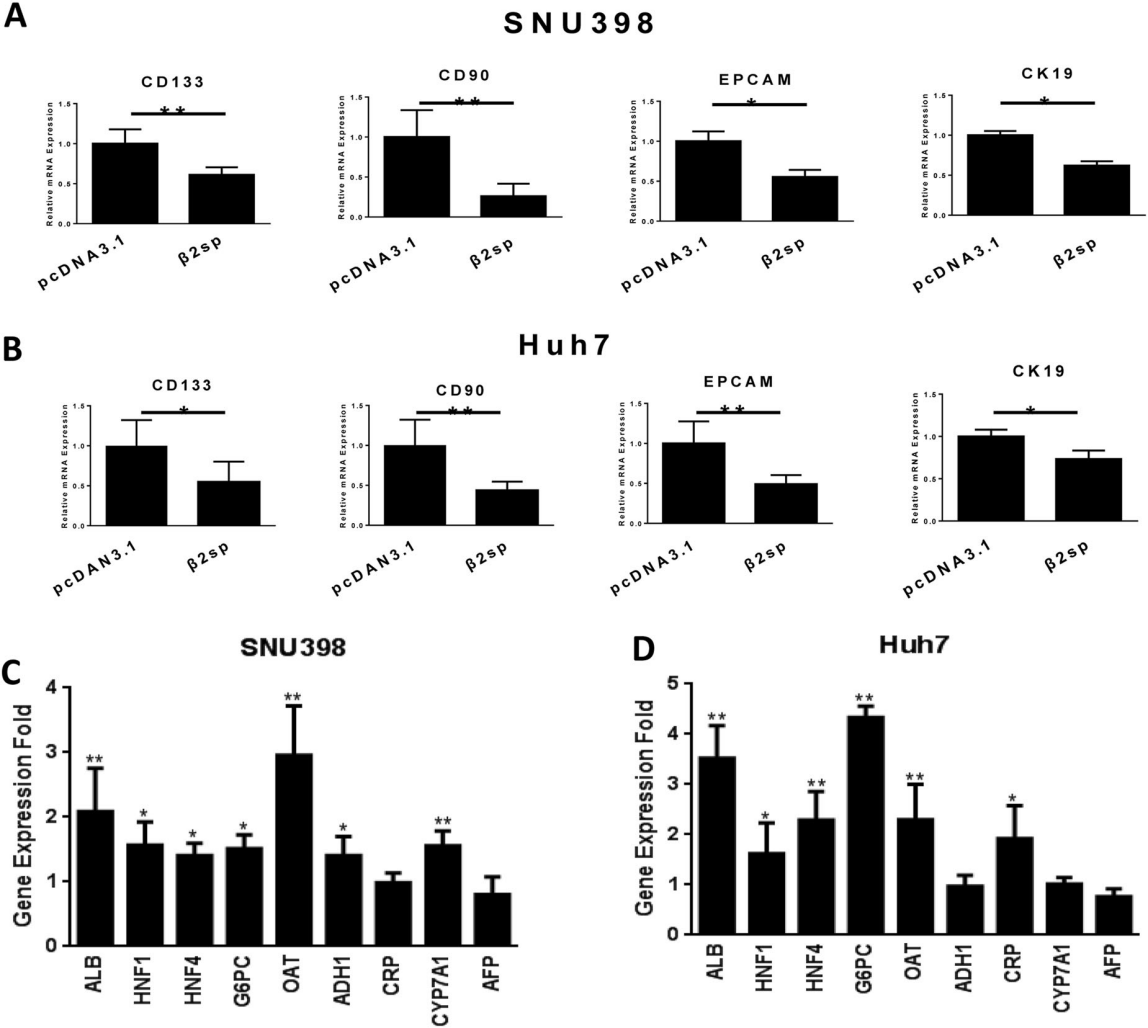
β2 spectrin promoted the differentiation of hepatoma cells and repressed transcription of liver CSC markers in HCC cells. **a**, **b** The mRNA level of liver CSC markers (CD133, CD90, EpCAM, and CK19) was measured in HCC cells (SNU398 and Huh7 cells) transfected with β2SP by real-time RT–PCR. **c**, **d** The mRNA level of liver-specific genes (ALB, HNF1α, HNF4α, G6PC, OAT, ADH1, CRP, CYP7A1) and AFP was measured HCC cells transfected with β2SP by real-time RT–PCR. GAPDH was used as internal control

Considering the involvement of β2 spectrin in the generation of liver CSCs^15^, we investigated the potential regulatory effect of β 2 spectrin on the expression of liver CSC markers and liver-specific genes through qRT–PCR and western blot. As shown in Fig. 4a, b and Fig. S5, β 2 spectrin repressed the expression of liver CSC markers (CD133, CD90, EpCAM and CK19) in transfected HCC cells (SNU398 and Huh7). In addition, the results of qRT–PCR and western blot (Fig. 4c, d, Fig. S5) showed that β2SP delivery increased the expressions of a cluster of liver-specific genes at the transcriptional level and protein level. Nevertheless, the expression of differentiation-associated marker AFP was not remarkably reduced after the treatment of β 2 spectrin. All these demonstrated β2 spectrin-mediated differentiation in HCC cells in vitro.

### CD133^+^ Huh7 and EpCAM^+^ SNU-398 cells were tumor-initiating cells with CSC features

In order to isolate tumor-initiating cells (TICs) from HCC cells, we first evaluated the expression pattern of liver CSC markers (EpCAM, CD133, CD90) in SNU-398 and Huh7 cells (Fig. S6A and B). Then, we isolated EpCAM^+^ or CD133^+^ cells from SNU398 or Huh7 cells by magnetic-activating cell sorting (MACS) according to flow cytometric analysis. We successfully enriched EpCAM^+^ and CD133^+^ populations, with more than 80% purity in EpCAM^+^ cells and more than 90% purity in CD133^+^ after sorting (Fig. S6C and D). Finally, the properties of CD133^+^ Huh7 and EpCAM^+^ SNU398 cells were determined through spheroid formation, colony-formation assays, chemoresistance, invasion assays, and in vivo tumorigenicity assay. As shown in Fig. S7, MACS-isolated EpCAM^+^ SNU-398 cells displayed liver CSC-like traits including more spheroid formation of cultured cells, increased colony-formation ability, appearance of drug-resistant to some chemo-therapeutics, and enhanced invasion ability. More important, these cells possessed stronger tumorigenicity in NOD-SCID mice. Simultaneously, MACS-isolated CD133^+^ Huh7 cells possessed the features of CSCs (Fig. S8), which was consistent with previous studies^26,27^.

### β 2 spectrin repressed cancer stem cell-like properties in tumor-initiating cells

**Fig. 5:**
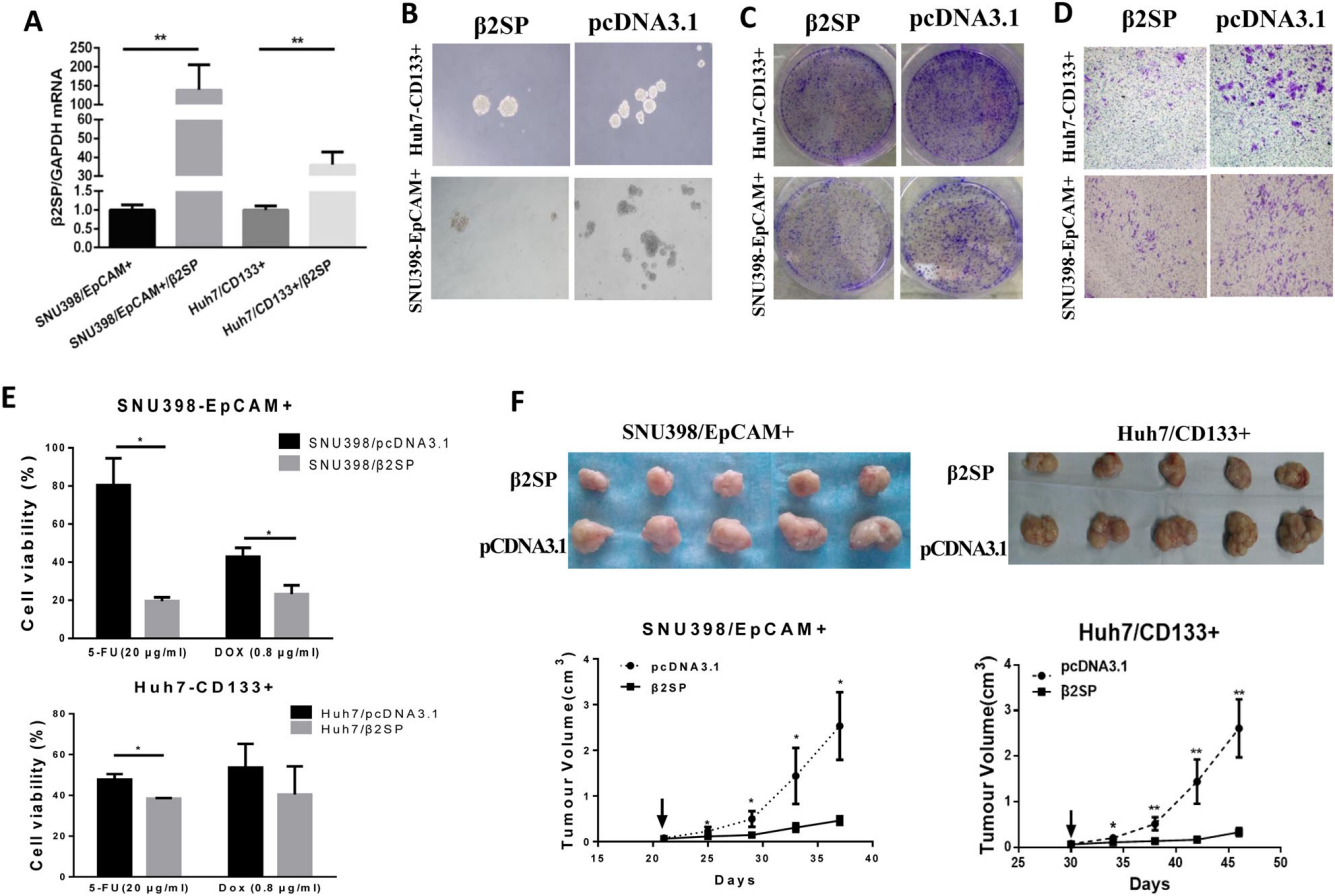
β2 spectrin repressed the properties of liver CSCs in liver tumor-initiating cells (T-ICs). The effect of β 2 spectrin on the properties of liver CSCs was evaluated by spheroid formation, colony formation, maltrigel invasion assay, chemo-resistant potential, and tumorigenicity in NOD/SCID mice. **a** qRT–PCR results showed that efficient β2SP expression in liver CSCs after β2SP cDNA transfection; **b** representative images of spheroid formation assay in liver CSCs transfected with the vector expressing β2 spectrin; **c** representative images of colony-formation assay in liver CSCs transfected with the vector expressing β2 spectrin; **d** representative images of Maltrigel invasion assay in liver CSCs transfected with the vector expressing β 2 spectrin; **e** CCK8 assay was used to compare cell viability between β2 spectrin- and control-transfected liver CSCs after treatment with 5-FU and DOX, respectively; **f** Representative images of xenograft tumors in NOD/SCID mice after the treatment. The tumor growth curves of each group of mice were summarized. The vectors expressing β2SP were administered directly into the tumor every 3 days for 5 times when the tumor approximately reached 5 mm diameter; arrow represented the time when the mice received first administration of the vector expressing β2 spectrin cDNA. All the data were means ± SEM of three independent experiments (**P < 0.01, *P < 0.05)

To answer the question whether β2SP repressed CSC-like properties in TICs, β2SP were transduced into TICs, and then in vitro and in vivo functional assays were performed. Firstly, we demonstrated showed the mRNA level of β2SP expression was significantly lower in liver CSCs (CD133^+^ Huh7 or EpCAM^+^ SNU-398 cells; Fig. S9). And then liver CSCs were transfected with the plasmids encoding β2SP cDNA because efficient lentivirus or adenovirus carrying β 2 spectrin cDNA (6.5 kb) were not prepared successfully. qRT–PCR results showed that efficient β2SP expression in EpCAM^+^ SNU398 and CD133^+^ Huh7 cells after β2SP cDNA transfection (Fig. 5a). Then, the effects of β2SP on the properties of CSCs were evaluated through functional assays containing spheroid formation, colony-formation assays, invasion assay, chemoresistance, and in vivo tumorigenicity assay. Spheroid formation assay found that β2 spectrin overexpression remarkably reduced sphere formation in CSCs (CD133^+^ Huh7 and EpCAM^+^ SNU-398 cells; Fig.5b, Fig. S10A). Colony-formation assay demonstrated that β2 spectrin reduced the frequency of colony formation in liver CSCs (Fig. 5c, Fig. S10B). Matrigel invasion assay revealed that *in vitro* metastatic abilities were inhibited in liver TICs after transfection with β2SP cDNA (Fig. 5d, Fig. S10C). Chemo-resistant assays showed that β2 spectrin increased the chemosensitivity of TICs to 5-Fu and Dox (Fig.5e). More important, the mice which had received administration of β2SP cDNA showed a marked suppression of xenograft tumors growth compared with the control (pcDNA3.1), which was revealed by gross morphology and growth curves (Fig. 5f). All these data demonstrated that β2 spectrin overexpression repressed cancer stem cell-like properties in liver tumor-initiating cells.

### β2 spectrin repressed transcription of liver CSC markers through β-Catenin in liver CSCs

**Fig. 6:**
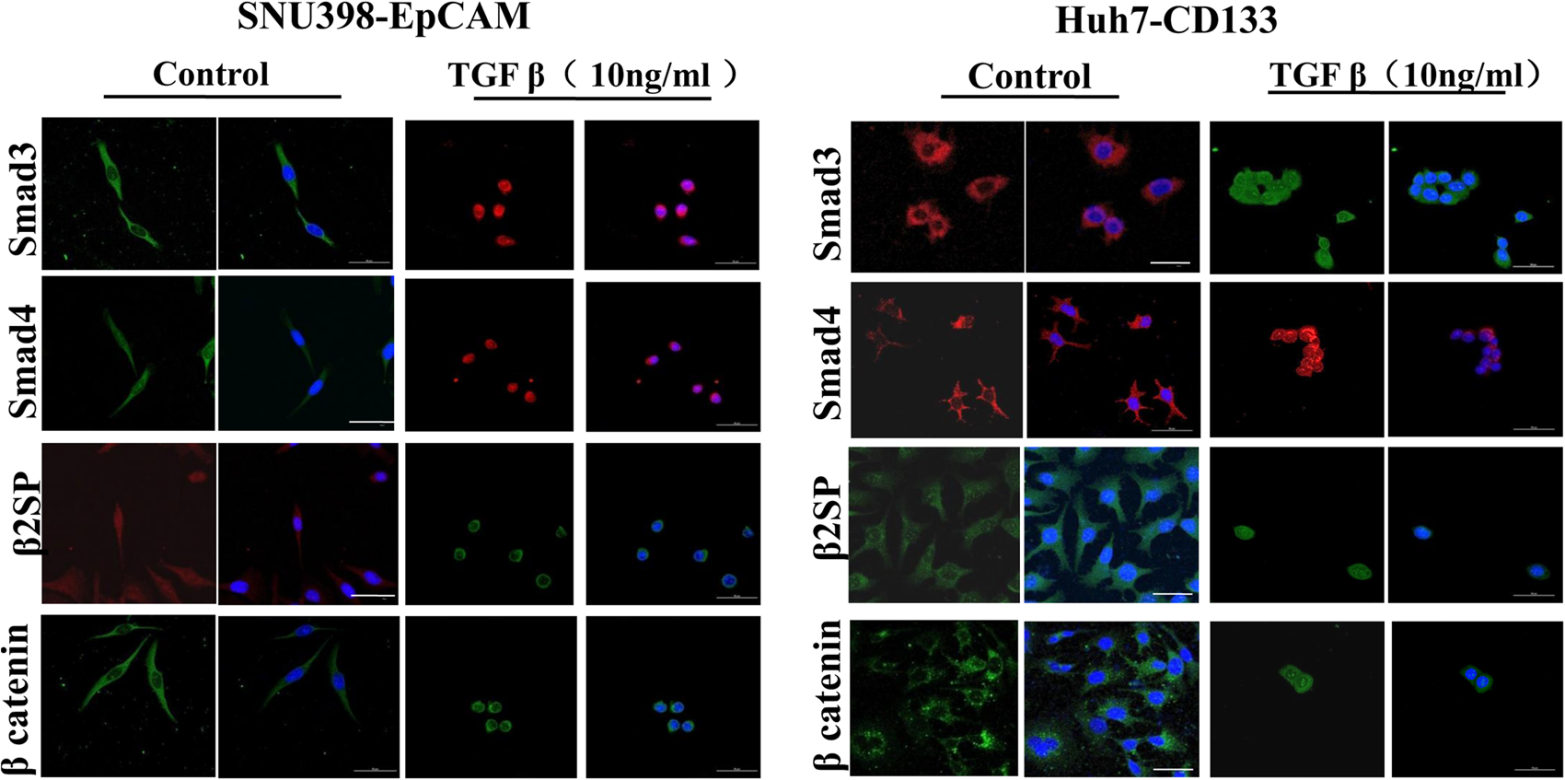
TGF-β1-stimulated nuclear translocalization of Smad3, Smad4, β2SP, β-catenin in liver CSCs. **a** Immunofluorescence staining investigated subcellular localization of Smad3, Smad4, β2SP, β-catenin in EpCAM^+^ SNU398 cells after TGF β stimulation. The results found that β2SP, β-Catenin and Smad3/4 translocated from cytoplasm to nucleus after TGF β1 stimulation for 6 h. **b** Immunofluorescence staining investigated subcellular localization of Smad3, Smad4, β2SP, β-catenin in CD133^+^ Huh7 cells after TGF β stimulation. Nuclear translocation ofβ2SP, β-Catenin and Smad3/4 was observed in CD133^+^ Huh7 cells after TGF β1 stimulation for 6 h

**Fig. 7:**
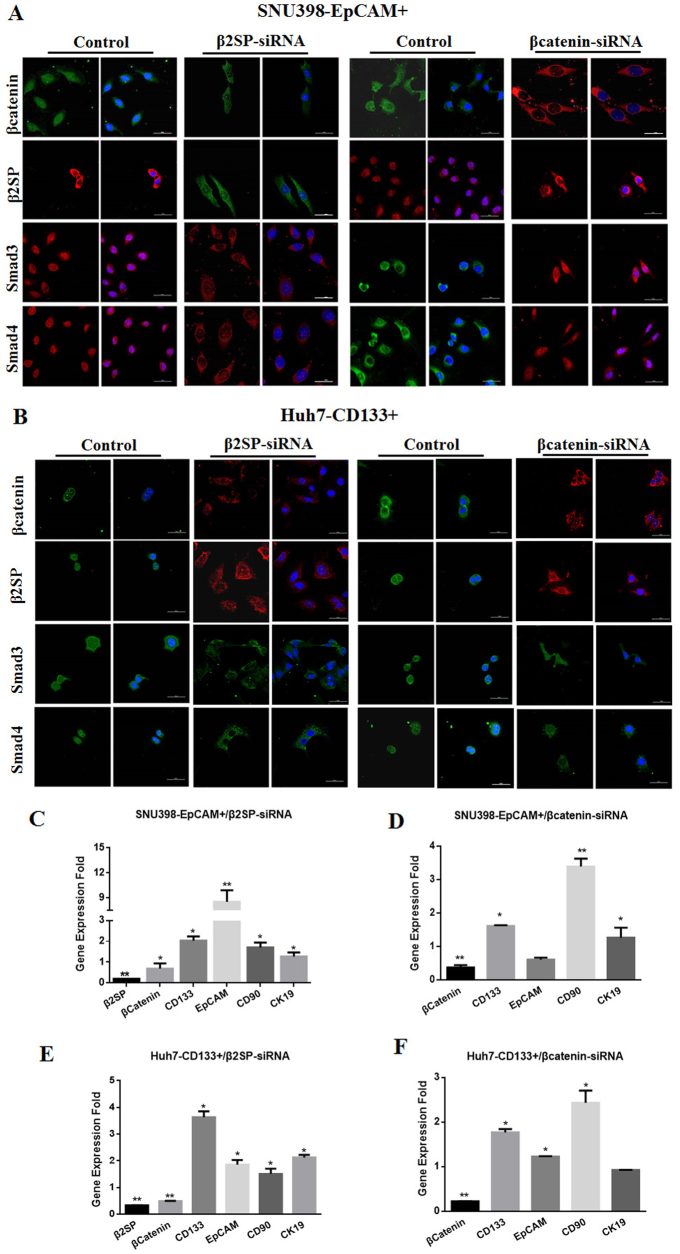
β2SP repressed the transcription of liver cancer stem cell markers through β-catenin in liver CSCs. **a** immunofluorescence staining determined subcellular localization of Smad3, Smad4, β2SP, β-catenin in EpCAM^+^ SNU398 cells after liver CSCs were transfected β2SP-siRNA and β-catenin siRNA respectively. **b** immunofluorescence staining determined subcellular localization of Smad3, Smad4, β2SP, β-catenin in CD133^+^ Huh7 cells after liver CSCs were transfected β2SP-siRNA and β-catenin siRNA respectively. **c** qRT–PCR demonstrated that liver CSCs markers increased in EpCAM^+^SNU398 cells with the transfection of β2SP-siRNA. In addition, downregulation of β-catenin mRNA was observed in liver CSCs after the treatment of β2SP-siRNA; **d** qRT–PCR showed the up-regulation of liver CSCs markers and downregulation of β-catenin in EpCAM^+^SNU398 cells transfected with β-catenin siRNA; **e** qRT–PCR demonstrated that up-regulation of liver CSCs markers in CD133^+^ Huh7 cells transfected with β2SP-siRNA; **f** qRT–PCR demonstrated that liver CSCs markers increased in CD133^+^ Huh7 cells with the transfection of β-catenin siRNA. GAPDH was used as internal control. All the data were means ± SEM of three independent experiments (**P < 0.01, *P < 0.05)

Wnt/β-catenin signaling is involved in several processes including embryonic development, cell fate determination, proliferation, polarity, migration, and stem cell maintenance^28^. Previous studies demonstrated that activation of β-catenin was sufficient for malignant transformation of liver progenitor cells, which contributed to the development of HCC^29–34^. In view of this, we investigated the involvement between β2SP and β-catenin in liver CSCs. Since β2SP was an adaptor of Smad3/4 complex in TGFβ/Smad signaling pathway, subcellular localization of Smad3/4 and β2SP was determined in the beginning. We found that Smad3, Smad4 and β2 Spectrin moved into the nucleus upon the stimulation of TGFβ1 in liver CSCs (CD133^+^ Huh7 and EpCAM^+^ SNU398 cells) (Fig. 6). In addition, translocation of β-catenin into nucleus was observed in liver CSCs upon the treatment of TGF β1. Then, the expression of β2SP and β-catenin was determined in liver CSCs transfected with siRNAs against β2SP and β-catenin. qRT–PCR (Fig. 7) demonstrated knockdown of β2 spectrin and β-catenin in EpCAM^+^ SNU-398 cells transfected with siRNA. Interestingly, downregulation of β-catenin was observed in EpCAM^+^ SNU-398 cells transfected with β2SP siRNA (Fig. 7, Fig. S11). To confirm this finding, subcellular localization of β2SP, Smad3/4, β-catenin was examined in liver CSCs transfected with siRNA against β2SP and β-catenin. In TGFβ1-stimulated EpCAM^+^ SNU398 cells transfected with β2SP siRNA, immunofluorescence results revealed that β2SP, Smad3, Smad4, β-catenin were localized in the cytoplasm, which resembled wild type in subcellular localization (Fig. 7a). On the other hand, cytoplasmic localization of β-catenin and nuclear localization Smad3, Smad4 and β2SP were observed in β-catenin siRNA-treated EpCAM^+^ SNU398 cells. Simultaneously, similar pattern was also shown in CD133^+^ Huh7 cells transfected with siRNA against β2 spectrin or β-catenin (Fig. 7b). These indicated TGFβ/Smad signaling stimulated the activation of β-catenin in liver CSCs. Finally, the expression of liver CSC markers and liver-specific marker genes was determined in liver CSCs transfected with siRNA against β2 spectrin or β-catenin through qRT–PCR. As shown in Fig. 7, the transcripts of liver CSC markers (CD133, CD90, EpCAM, and CK19) increased in liver CSCs transfected with β2 spectrin or β-catenin siRNA. Unfortunately, the effect of β2SP or β-catenin siRNA on the expression of liver-specific genes was not evaluated because mRNA level of liver-specific genes was relative low in liver CSCs. These data indicated β2 spectrin repressed transcriptional activity of liver CSCs markers through β-catenin in liver CSCs.

## Discussion

β II-spectrin is a crucial Smad3/4 adaptor and transcriptional cofactor during TGF-β signal transduction^12,35,36^. Previous studies demonstrated that dysfunction of TGFβ signaling result from the loss of β2SP contributed to the development of HCC^14,15,17,37^. In this study, we demonstrated that β2SP overexpression restrained the proliferation of HCC cells and abolished their tumorigenicity in vitro. In view of these, we investigated the possible mechanisms by which loss of β2SP contributed to tumorigenesis. Hepatocyte dedifferentiation is a key cellular event during hepatocarcinogenesis, which is featured by an alteration of morphology and loss of hepatic function. Our studies showed that levels of β2SP expression were closely associated with the differentiation status of hepatocytes in DEN-triggered rat model of hepatocarcinogenesis and clinical specimens. Next, we introduced β2 spectrin into HCC cell lines, and demonstrated that β2SP promoted the differentiation of HCC cells and suppressed the growth of HCC cells in vitro. All these indicated that the inhibitive effect of β2SP on the development of HCC was attained by inducing HCC cells into a differentiated status.

There is growing evidence that cells within HCC exhibit functional heterogeneity and include a subpopulation of cancer cells referred to as cancer stem cells (CSCs) or tumor-initiating cells (TICs)^9,10^. CSCs are capable of extensive proliferation, self-renewal, and increased frequency of tumor formation. Liver CSCs have recently been identified in HCC and are known to drive tumor initiation, self-renewal, chemoresistance, and metastasis^11,38^. A number of liver CSC markers have been identified, including CD133, CD90, EpCAM, CD44, CK19, OV6^11,38–40^. In our study, we isolated EpCAM^+^ or CD133^+^ from SNU-398 or Huh7 cells using MACS. Further studies demonstrated EpCAM^+^ SNU398 and CD133^+^ Huh7 cells displayed the capacity of liver CSCs. To test the effect of β2 spectrin on CSC-like properties, functional assays were applied to determine CSC properties of CD133^+^ Huh7 and EpCAM^+^ SNU398 cells. The results demonstrated that efficient transduction of β2 spectrin into liver CSCs resulted in a reduction in colony-formation ability, spheroid formation capacity, invasive activity, and chemoresistance properties, tumorigenesis of CSCs in vivo. All these revealed that β2 spectrin repressed CSC properties. Differentiation therapy represents a novel promising therapeutic strategy for human HCC treatment. Our data demonstrated that β2 spectrin induced the differentiation of liver CSCs, and then repressed the CSCs properties in liver tumor-initiating cells. Therefore, the restoration of β2 spectrin in liver CSCs might be developed as a new promising candidate for the treatment of HCC.

Some studies explored molecular mechanism underlying the role of β2 spectrin in pathogenesis of HCC. Bake et al.^41^ demonstrated β2SP deficiency led to CDK4 activation and contributed to dysregulation of the cell cycle and the formation of HCCs. Thenappan et al.^20^ showed that β2SP loss may increase susceptibility to DNA damage, impair cell cycle progression, delayed liver regeneration, and ultimately lead to hepatocellular cancer. While, our studies showed β2SP promoted the differentiation of HCC cells and repressed transcription of liver CSC markers. More importantly, β2SP suppressed the transcriptional activity of liver CSC markers in liver CSCs. It indicated that β2SP-mediated differentiation repressed the properties of liver CSC. Then, we investigated the involvement between β2 spectrin and β-catenin in liver CSCs. Activation of TGF β/Smad signaling stimulated the activation of β-catenin in liver CSCs, and blockage of TGF β/Smad signaling was not observed in CSCs treated with β-catenin siRNA. These revealed that β2 spectrin repressed the features of liver CSC markers through β-catenin. Zhi et al. demonstrated that loss of β2 spectrin activated Wnt signaling in HCC cell lines, which promotes acquisition of stem cell-like features, and ultimately contributes to malignant tumor progression^17^. Although some similarity, there were some fundamental differences between their study and our research. In methods, Zhi’s research used HCC cell line and β2SP knockout mice, our study used liver cancer stem cells, DEN-induced HCC model and clinical species. In signaling pathway, Zhi’s research showed that loss of β2SP activates Wnt signaling and increased β-catenin nuclear localization; our study showed that loss of β2SP suppressed nuclear translocalization of β-catenin in liver cancer stem cells. Liver CSCs used in our study and HCC cells in previous study^17^ may result in the disparity in signaling pathway. The difference in the methods and mechanism gave rise to the disparity in the conclusions.

In conclusion, dedifferentiation is a distinctive feature of HCC progression, our data suggested that β2 spectrin induced the differentiation of liver cancer stem cells, and then repressed the CSCs properties in liver tumor-initiating cells. Strategies to restore its levels and activities could be a novel strategy for HCC prevention and differentiation therapy.

## Electronic supplementary material

clean supplementary data(DOC 2585 kb)
