# Supplementary material for: β2 spectrin-mediated differentiation repressed the properties of liver cancer stem cells through β-catenin
Source: Cell Death Dis. 2018 Mar 19;9(4):424. doi: 10.1038/s41419-018-0456-6 (PMC5859291; doi:10.1038/s41419-018-0456-6)
Supplement: Supplementary file 1 — clean supplementary data(DOC 2585 kb) [file 41419_2018_456_MOESM1_ESM.doc]

**Figure S1**


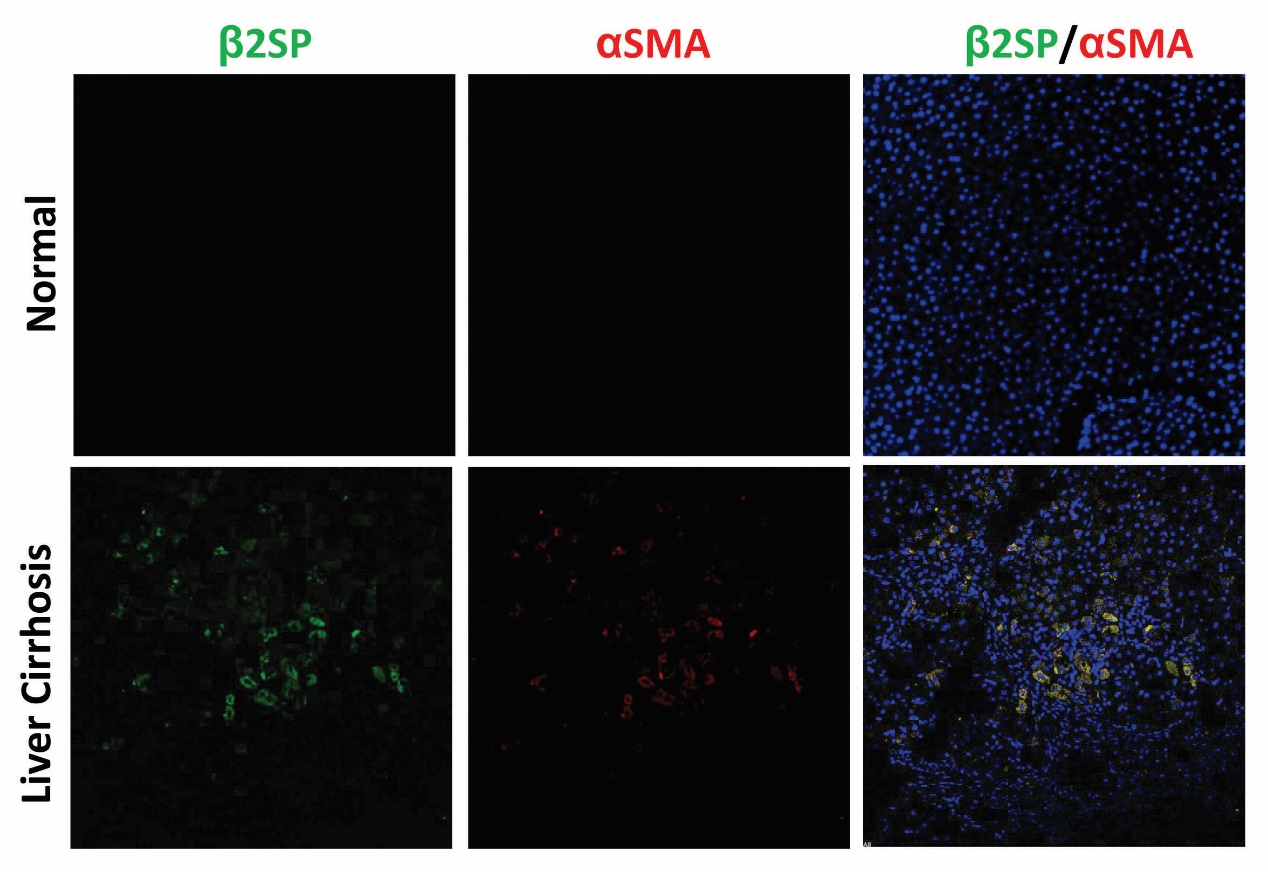


FigS1. Liver section were obtained from the patients with liver cirrhosis, and then coimmunostaining for α-smooth muscle actin (αSMA) (red) and β 2 spectrin (green) was performed. α-smooth muscle actin is a marker of activated hepatic stellate cells. Colocalization of β2SP (green)and α-SMA (red) was observed in human cirrhotic tissues, which indicated overexpression of β 2 spectrin in activated HSCs. Magnification 200×.

**Figure S2**


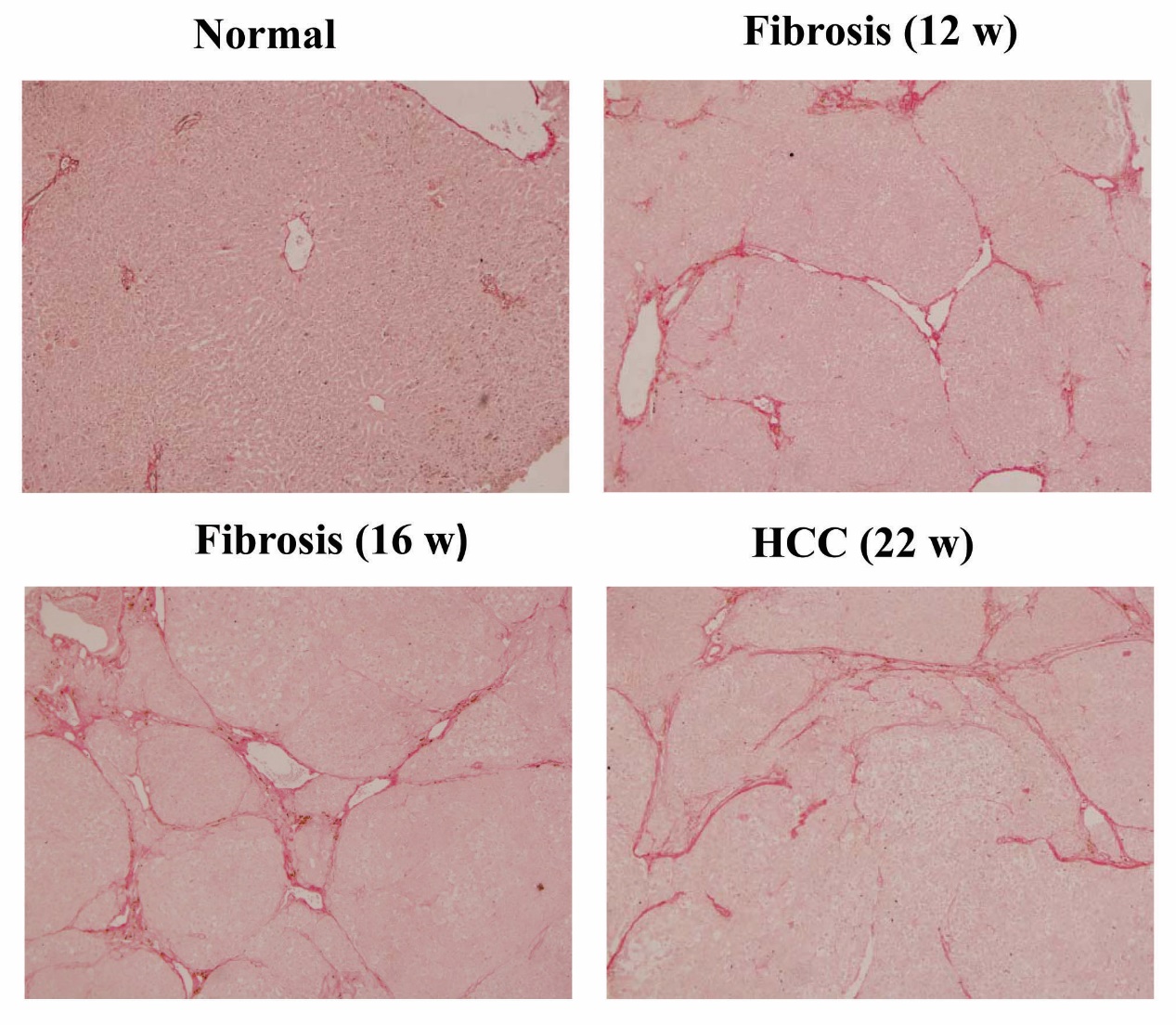


Fig S2. In diethylnitrosamine (DEN)-induced model of liver fibrosis and hepatocellular carcinoma(HCC), Wistar rats were killed at week 12 (fibrosis),week 16(fibrosis) or week 22(hepatocellular carcinoma) after DEN administration. Liver tissues were collected and subjected to Sirius red staining. Magnification 100×.

**Figure S3**


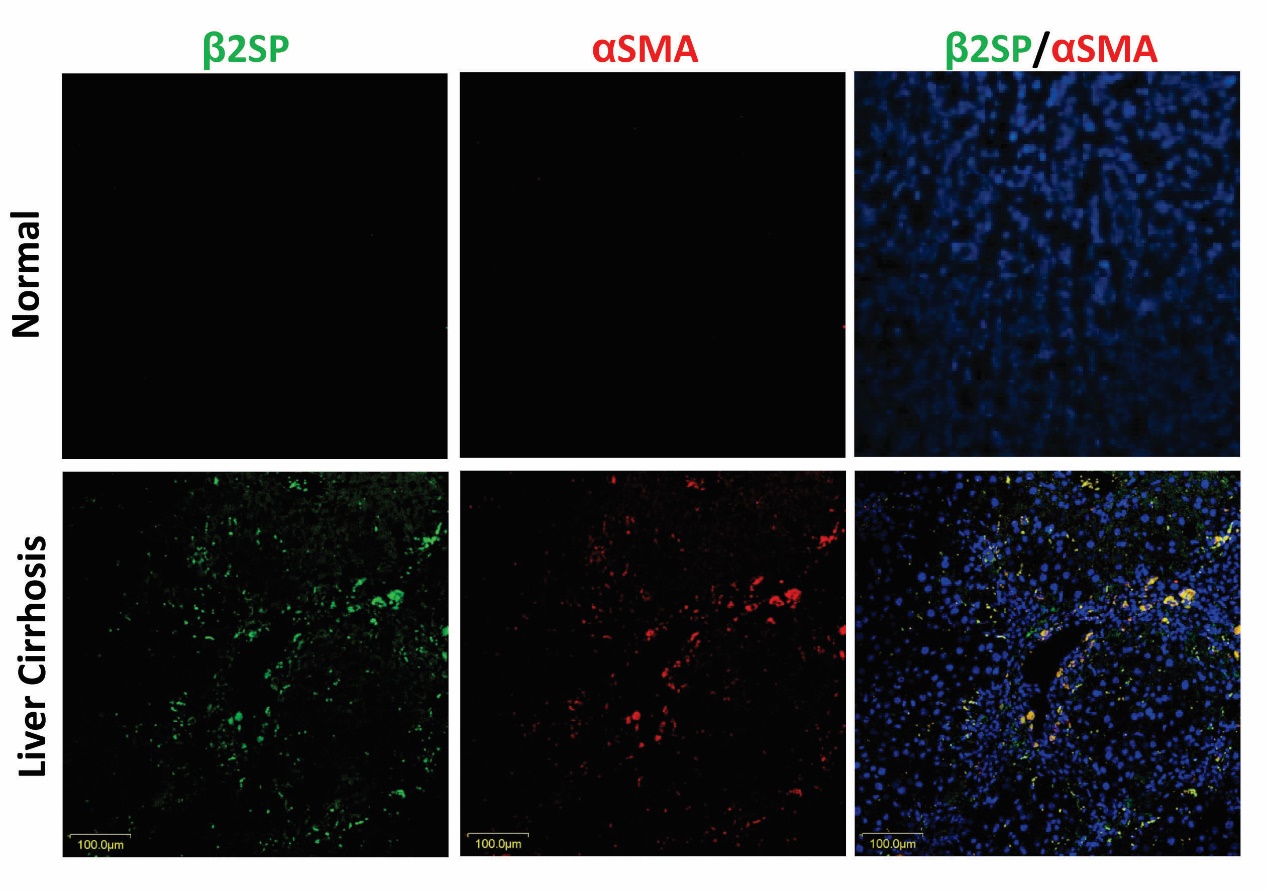


Fig S3: To determine whether activated stellate cells express β 2 spectrin in rat model of liver fibrosis, coimmunostaining for α-smooth muscle actin (αSMA) (red) and β2 spectrin (green) was performed on liver section obtained from DEN-induced model of liver fibrosis. The results showed that β 2 spectrin became overexpressed predominantly in activated HSCs. Magnification 200×.

**Figure S4**


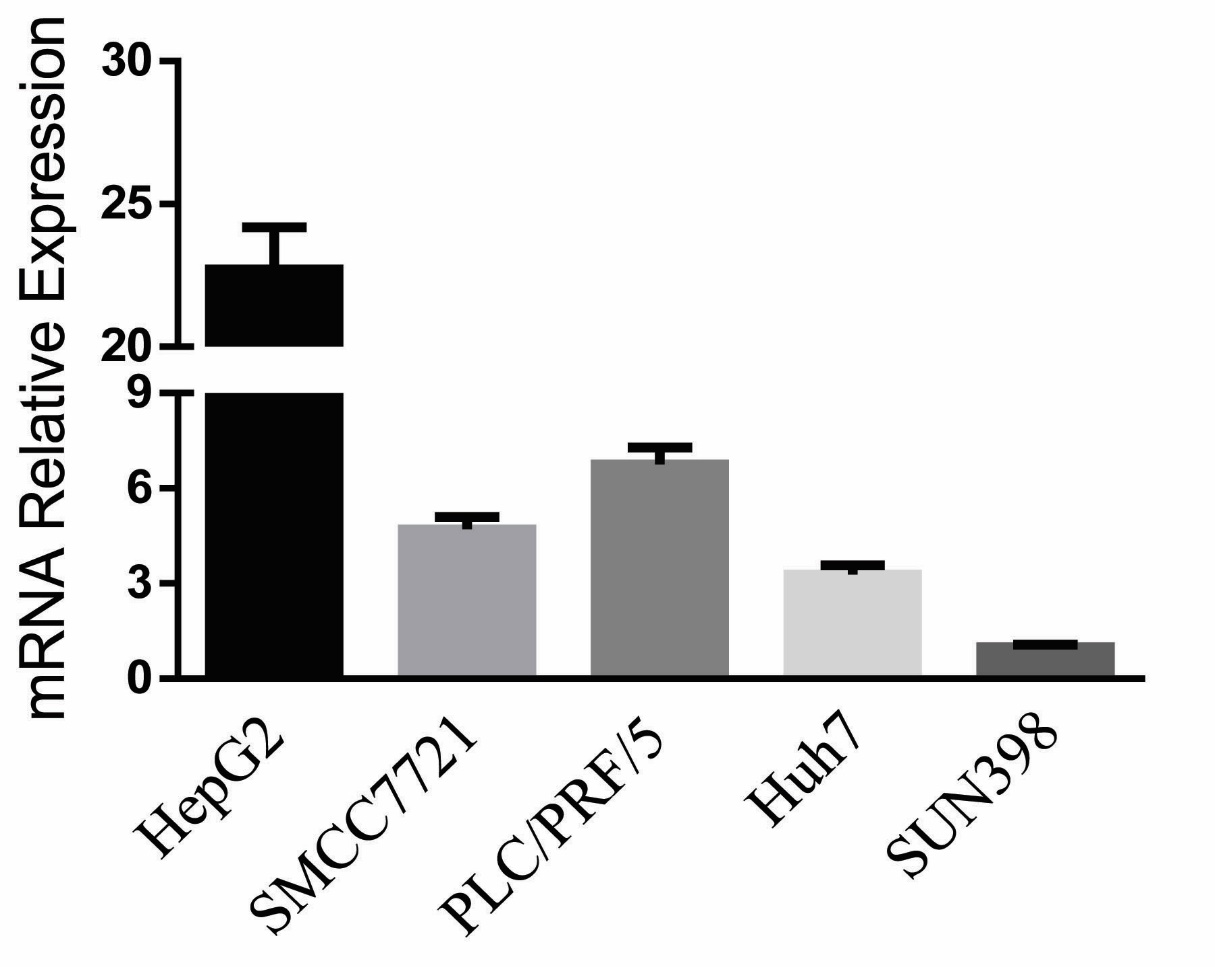


Fig. S4 Quantitative RT-PCR determined mRNA level of β2SP in different human HCC cell lines (HepG2, SMCC7721, PLC/PRF/5, Huh7 and SUN398). The result showed low level of β2SP mRNA transcripts in Huh7and SUN398 cells.

**Figure S5**

**
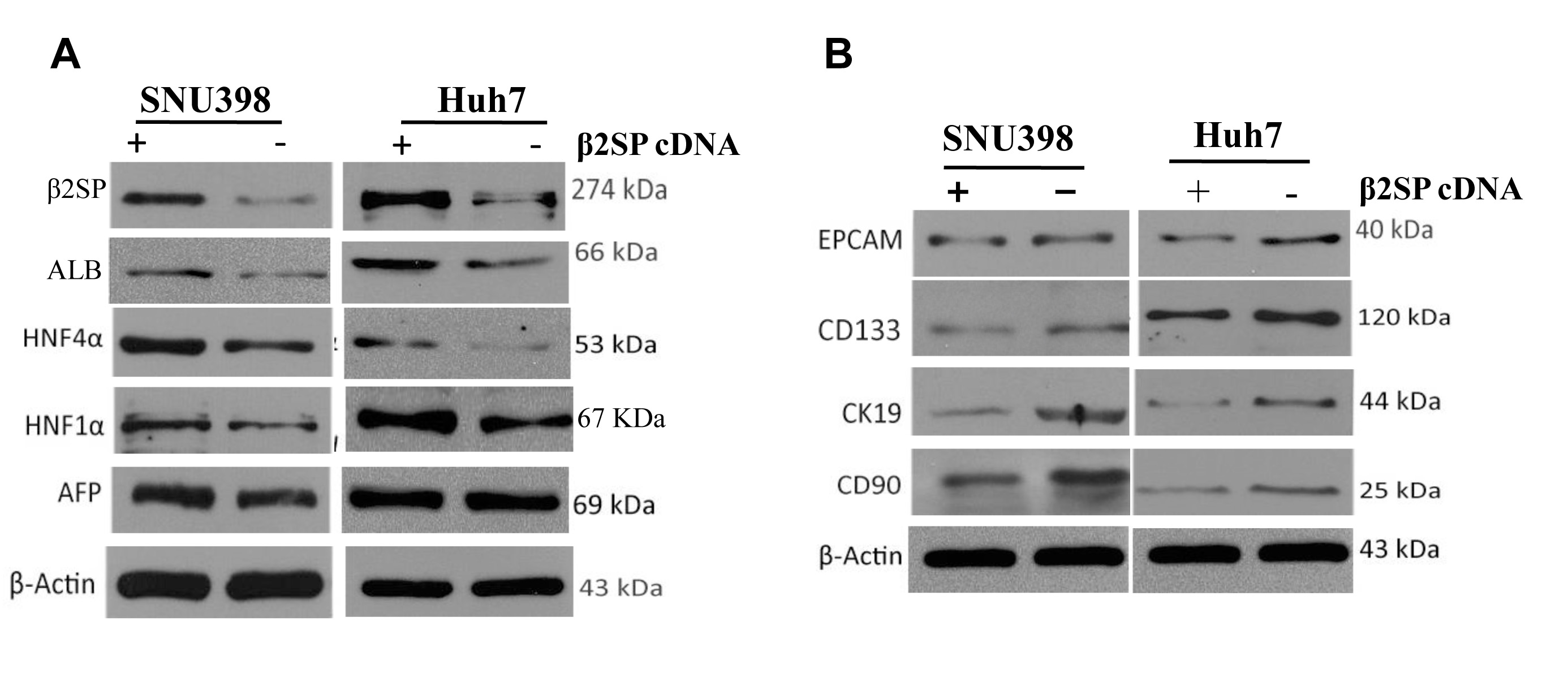
**

Fig S5 Western blot analysis determined the expression of liver CSC markers and liver-specific genes in HCC cells transfected with the vector expressing β2SP cDNA or the control.

1. Western blot analysis determined the expression of liver-specific genes in SNU398 and Huh7 cell lines after transfected with the vector expressing β2SP cDNA or the control.
2. Western blot analysis determined the expression of liver CSC makers in SNU398 and Huh7 cell linesafter transfected with the vector expressing β2SP cDNA or the control.

**Fig. S6**


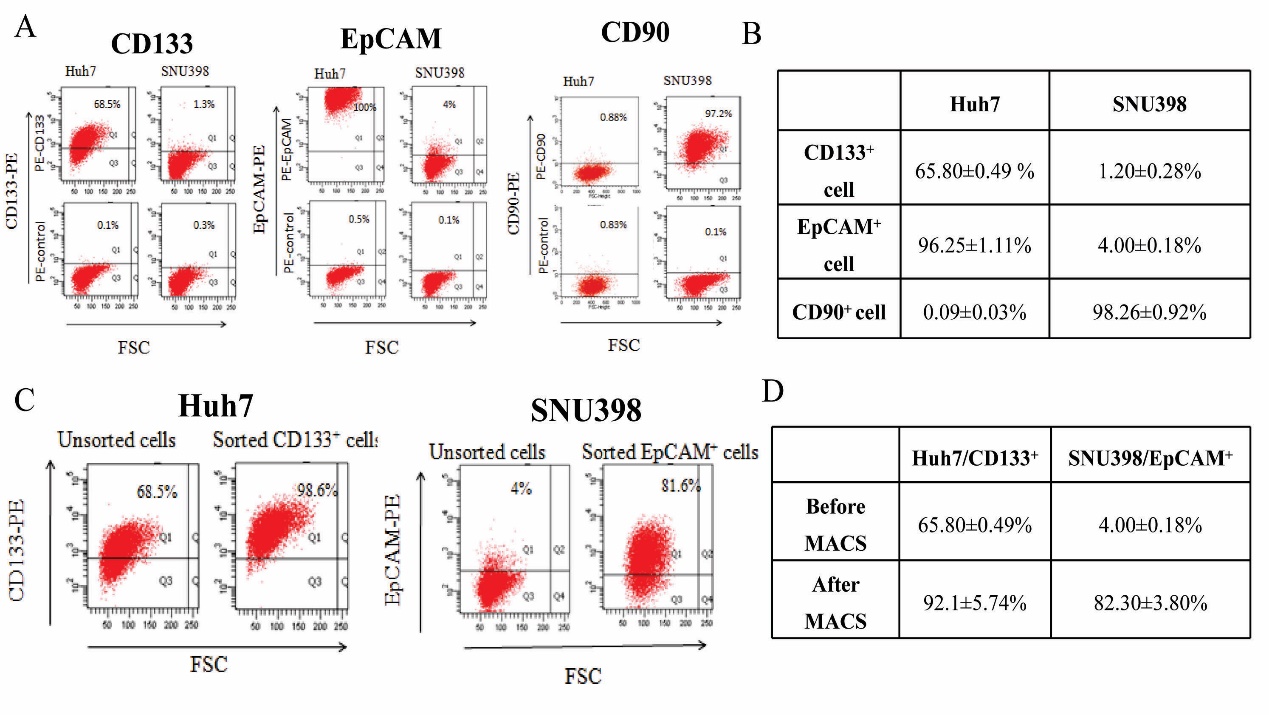


Fig .S6 flow cytometric analysis of liver CSCs markers in Huh7 and SNU398 cells before and after [magnetic-activating cell sorting](https://www.bing.com/academic/profile?id=762e13ed1091af93429c80c9af761eea&encoded=0&v=paper_preview&mkt=zh-cn) (MACS).

1. representative flow cytometric histogram depicted the surface expression of CD133, EpCAM, CD 90 in Huh7 and SNU398 cells;
2. the data obtained by triple separate experiments showed the mean expression of CD133, EpCAM, CD90 in Huh7 and SNU398 cells;
3. representative flow cytometric histogram detected the expression of CSCs markers in Huh7 and SNU398 cell before and after [magnetic bead cell sorting](https://www.bing.com/academic/profile?id=762e13ed1091af93429c80c9af761eea&encoded=0&v=paper_preview&mkt=zh-cn);
4. the data obtained by triple separate experiments showed the mean expression of CD133, CD90 in Huh7, SNU398 cell lines before and after MACS; the results showed that CD133^+^ Huh7 or EpCAM^+^ SNU398 cells were enriched by cell sorting.

**Figure.S7**
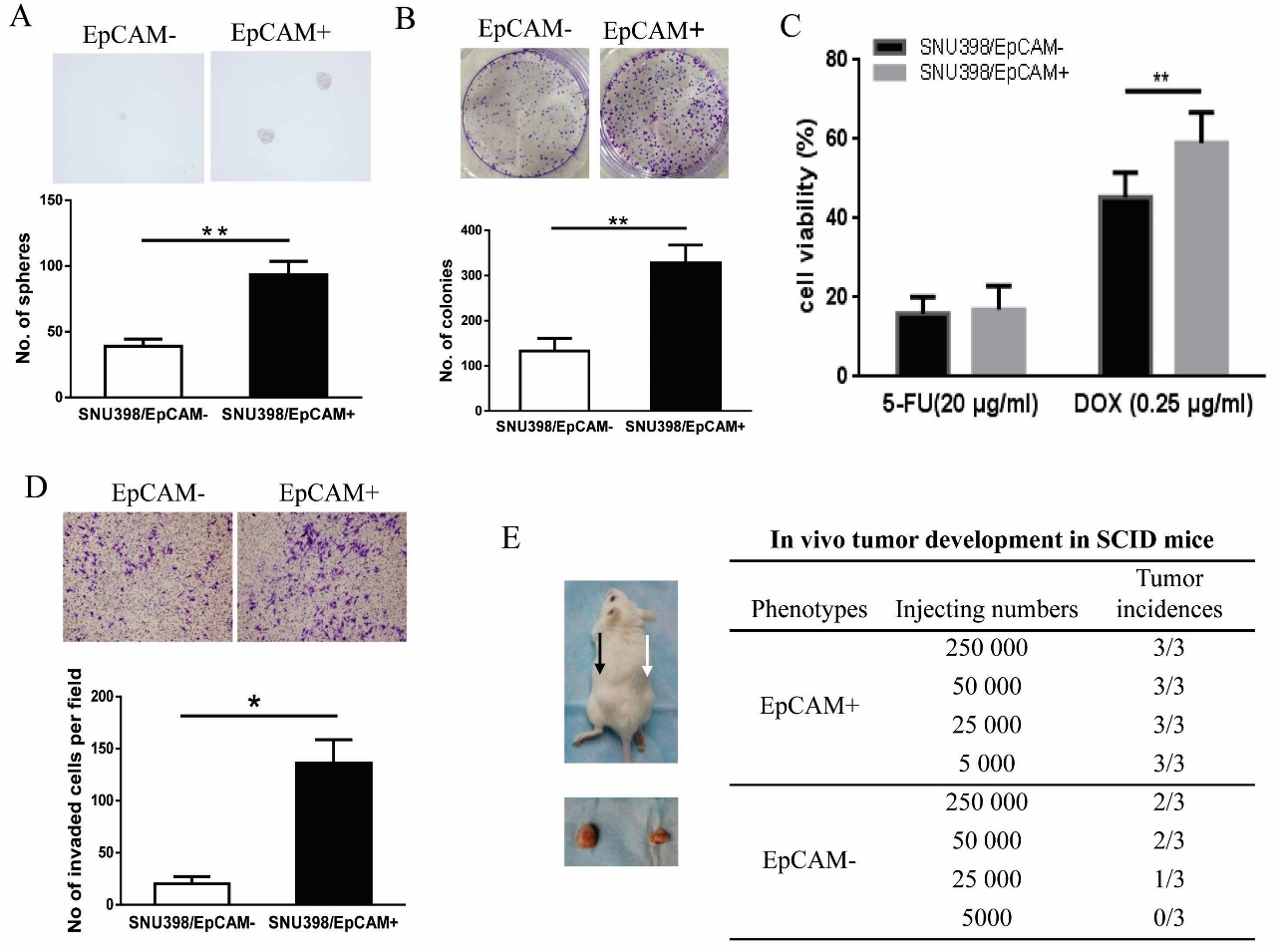


Fig S7: EpCAM^+^ SNU398 cells possessed the features of liver cancer stem cells. The features of liver cancer stem cells were determined through spheroid formation, colony formation assays, chemo-resistance, invasive activity and *in vivo* tumorigenicity assay.

1. Representative photographs of spheroids derived from EpCAM^+^ or EpCAM^-^SNU398 cells (*upper panel*) and spheroid formation experiments were performed in triplicate (Means ±SEM) (*lower panel*).
2. Representative photographs of colonies derived from EpCAM^+^ or EpCAM^-^SNU398 cells (*upper panel*) and colon formation experiments were performed in triplicate (Means ±SEM) (*lower panel*). Colon formation assay revealed that EpCAM^+^ cells formed colonies more efficiently than EpCAM^-^ cells.
3. Proliferation assay showed that EpCAM^+^ cells had higher cell viability than EpCAM^-^ cells after the treatment of chemotherapeutic drugs (0.25 μg/ml DOX ) using Cell Counting Kit-8 (CCK8);
4. the matrigel invasion assay demonstrated that EpCAM^+^ cells had more invasive potential than EpCAM^-^ cells;
5. *In vivo* tumorigenic ability of EpCAM^+^ or EpCAM^-^ cells was investigated in xenograft mice model. EpCAM^+^ or EpCAM^-^cells were subcutaneously injected into NOD/SCID mice, and the results revealed that xenograft tumors derived from the injection of EpCAM^+^ cells grew faster than those from EpCAM^-^ cells.

All the data were Means ±SEM of 3 independent experiments (** P <0.01, * P<0.05).

**Figure S8**


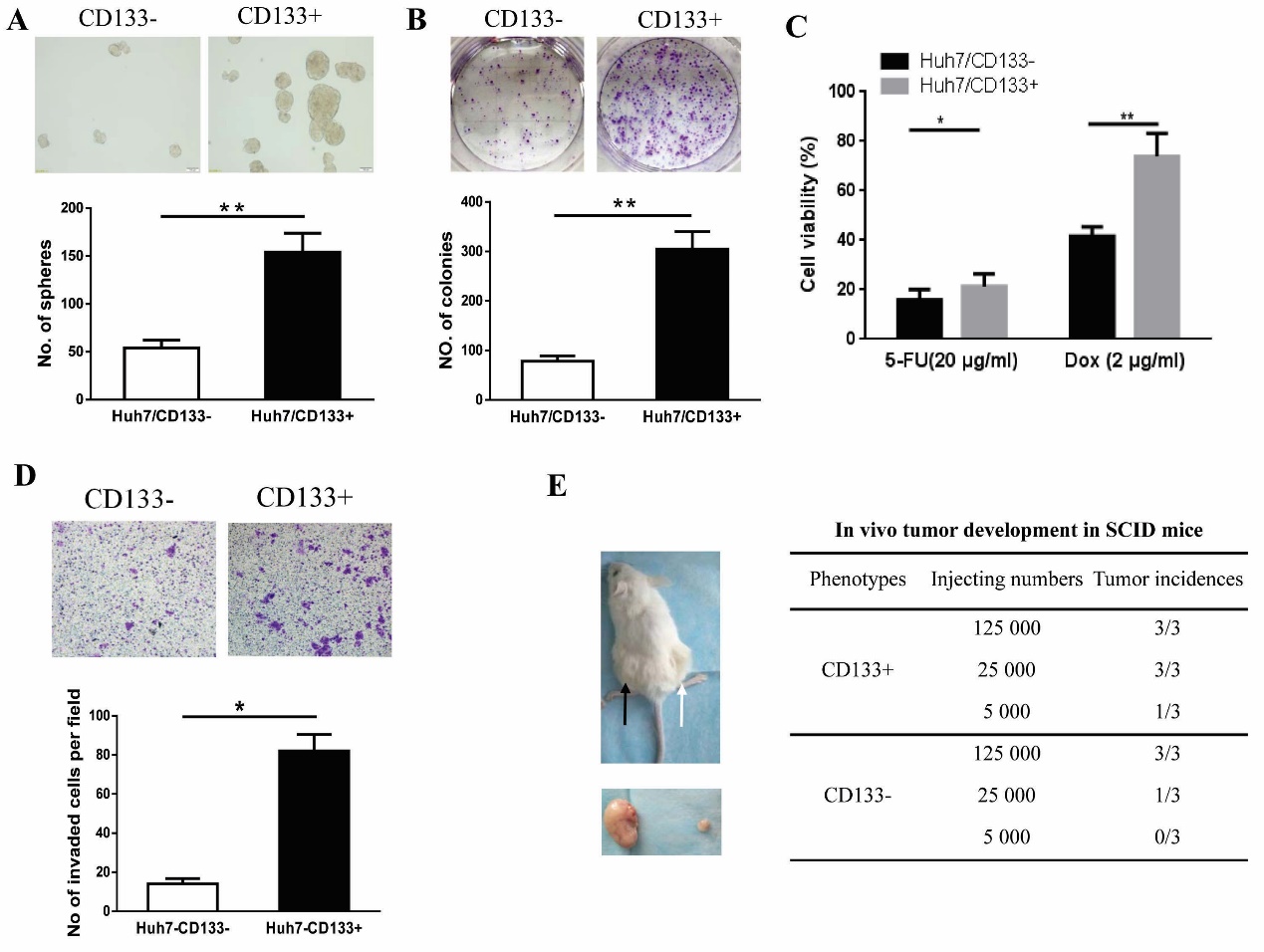


Fig S8: CD133^+^Huh7 cells possessed the features of cancer stem cells. The features of liver cancer stem cells were determined through spheroid formation, colony formation assays, chemo-resistance, invasive activity and *in vivo* tumorigenicity assay.

1. spheroid formation assay demonstrated CD133^+^ cells generated more spheres than CD133^-^ cells in serum-free medium;
2. Colon formation assay revealed that CD133^+^ cells formed colonies more efficiently than CD133^-^ cells.
3. Proliferation assay showed that CD133^+^ cells had higher cell viability than CD133^-^ cells after the treatment of chemotherapeutic drugs (20 μg/ml 5-FU and 2 μg/ml DOX) using CCK8;
4. the matrigel invasion assay demonstrated that CD133^+^ cells had more invasive potential than CD133^-^ cells;
5. *In vivo* tumorigenic ability of CD133^+^ or CD133^-^ cells was investigated in xenograft mice model. CD133^+^ or CD133^-^cells were subcutaneously injected into NOD/SCID mice, and the results revealed that xenograft tumors derived from the injection of CD133^+^ cells grew faster than those from CD133^-^ cells. All the data were Means ±SEM of 3 independent experiments (** P <0.01, * P<0.05)

**Figure S9**

**
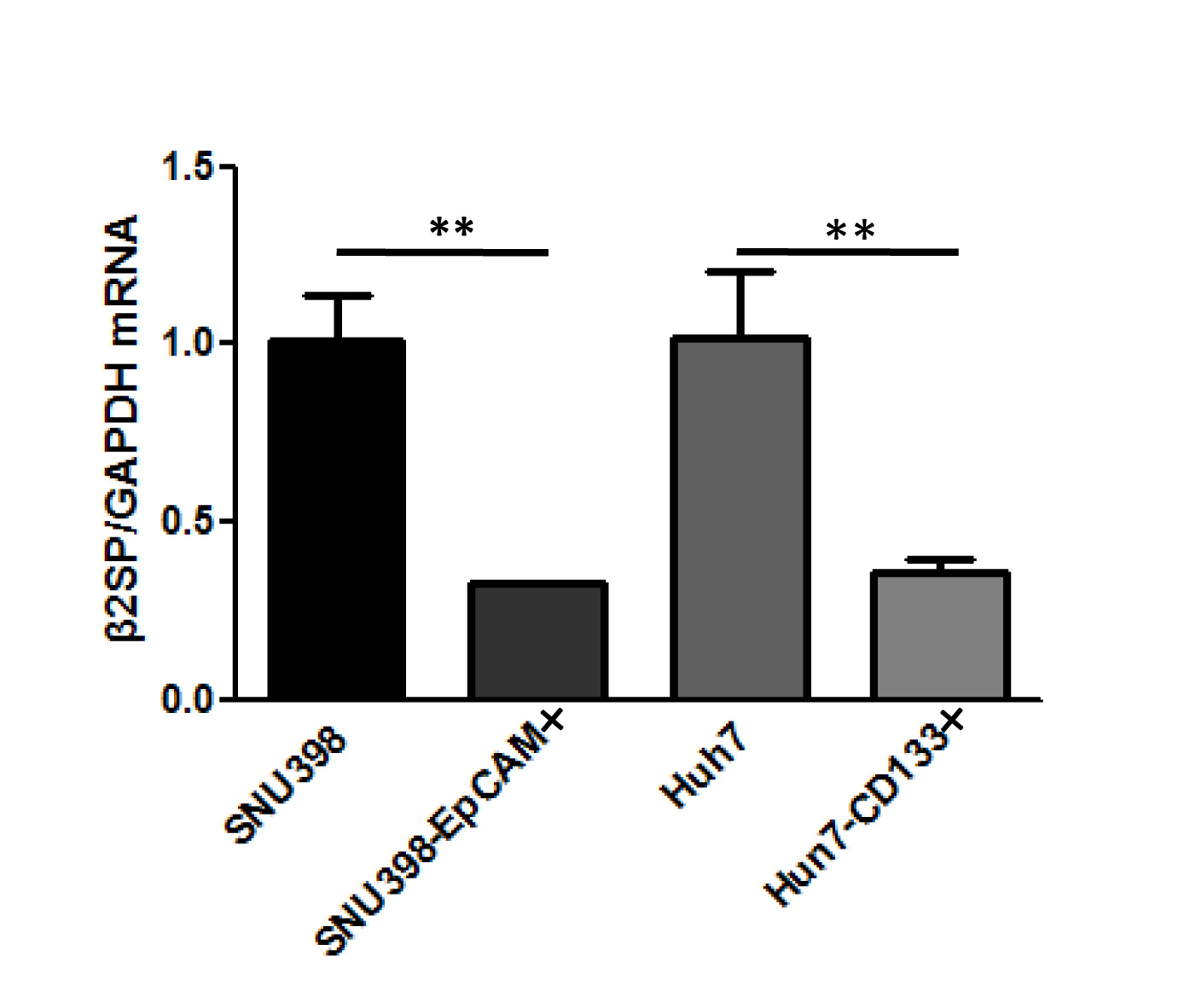
**

Fig. S9: Quantitative RT-PCR showed down-regulation of β2SP expression in liver cancer stem cells (EpCAM^+^ SNU398 and CD133^+^ Huh7 cells).

**Fig.S10**


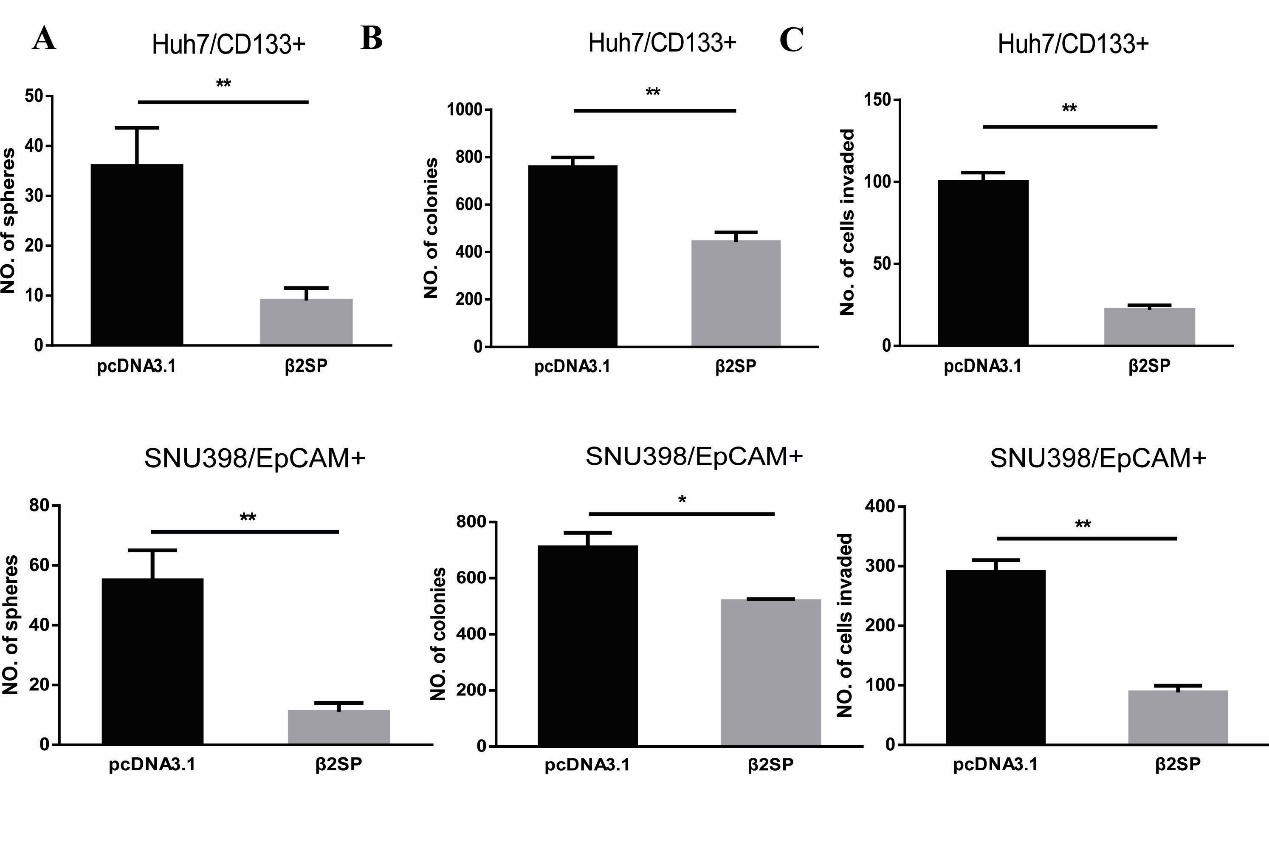


Fig S10: liver cancer stem cells (CD133^+^ Huh7 or EpCAM^+^ SNU398 cells) were transfected with the plasmids expressing β2SP cDNA, and *in vitro* functional assays containing spheroid formation, colony-formation, maltrigel invasion were performed. The results demonstrated β2SP suppressed CSC features *in vitro*.

1. In the sphere formation assay, the self-renewal ability was inhibited in sorted liver CSCs (CD133^+^ Huh7 or EpCAM^+^ SNU398 cells) transfected with the plasmids expressing β2SP cDNA.
2. Colon formation assay showed that colony-formation capacity was suppressed in liver CSCs (CD133^+^ Huh7 or EpCAM^+^ SNU398 cells) transfected with the plasmids expressing β2SP cDNA
3. The Matrigel invasion assay demonstrated that invasion ability was suppressed in liver CSCs (CD133^+^ Huh7 or EpCAM^+^ SNU398 cells) upon the transfection of β2SP cDNA.

All the data are Means ±SEM of 3 independent experiments (** P <0.01, * P<0.05).

**Figure S11**

**
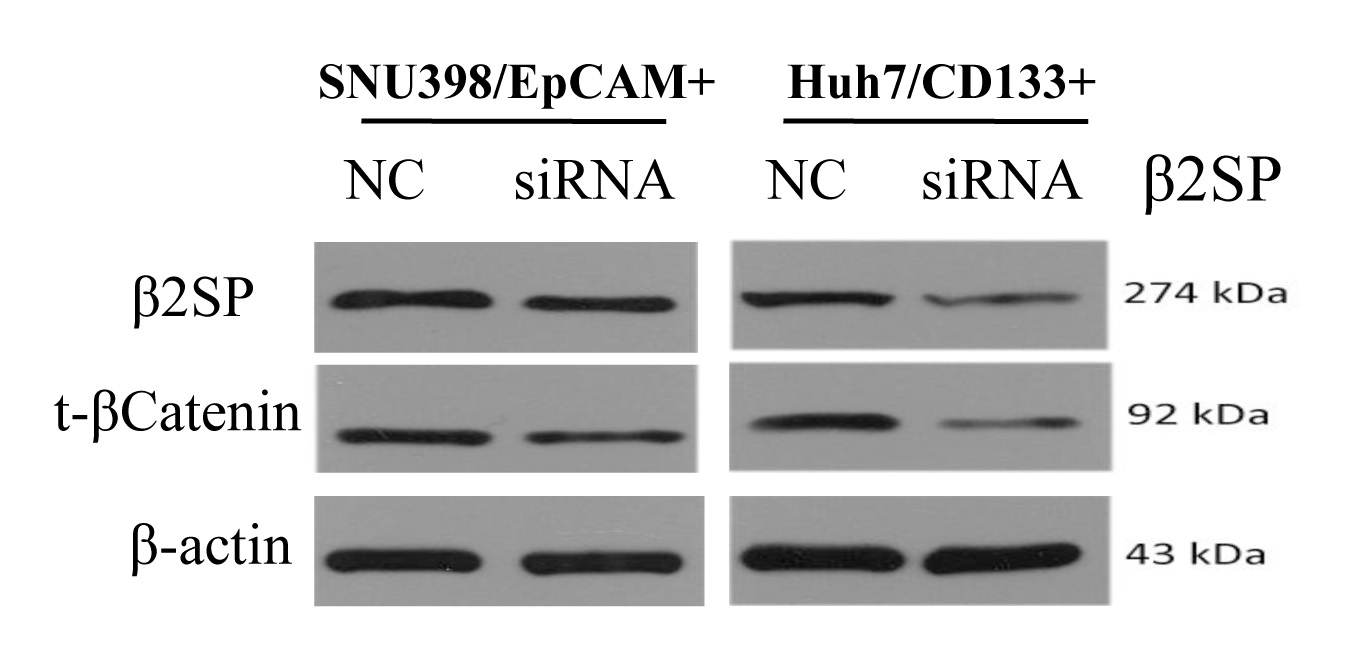
**

Fig S11: western blot showed that down-regulation of β 2SP and β catenin was observed in EpCAM^+^ SNU398 and CD133^+^ Huh7 cells transfected with β2SP siRNA.

**Figure S12**

**
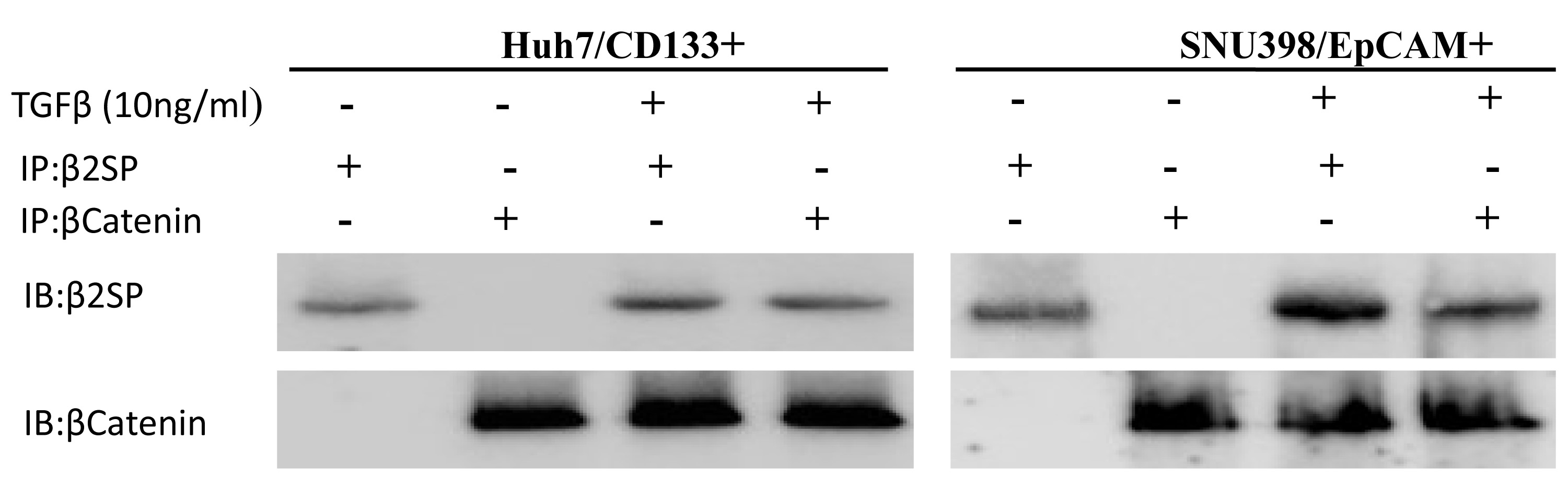
**

Fig.S12: Interaction of β2SP and β-catenin in liver cancer stem cells. Lysates was subjected to immunoprecipitation with antibody to β2SP or β-catenin; and followed by immunoblotted. Coimmunoprecipitation of β2SP with β-catenin was shown in the presence of TGFβ1.

**Materials and methods**

**Rat models of liver fibrosis and HCC**

80 male Wistar rats (aged 6-8 weeks)were purchased from Beijing Vital River Laboratory Animal Technology Co., Ltd. (Beijing, China).The rats weighing 200 g were injected intraperitoneally with N-nitrosodiethylamine (DEN) (N0756; Sigma-Aldrich, St. Louis, MO) or 0.9% NaCl(Control) at a dose of 50mg/kg once a week for 16 weeks. Rats were sacrificed randomly at 12,16, and 22 weeks after DEN administration. The protocol of animal treatment used in this study was approved by the institutional animal care and use committee.

**Histological examination**

Human liver cancer specimens (15 patients) and normal liver specimens (6 patients) were obtained from Department of Hepatobiliary Surgery, Union Hospital, Tongji Medical College (Wuhan, China). The rat model of liver fibrosis and HCC was induced by intraperitoneal injection of DEN. Five-micrometer-thick tissue sections of formalin-fixed, paraffin-embedded liver samples were stained with hematoxylin and eosin for standard histology. Sirius red staining and immunohistochemistry were performed following the routine protocol. Antibodies used were listed in supplementary Table 1.

**Cell culture**

HCC cell lines HepG2, SNU-398, PLC/PRF/5, Huh7were purchased from Cell Bank of Type Culture Collection of Chinese Academy of Sciences. HepG2, SMCC7721, PLC/PRF/5 and Huh7 cells were cultured in 10%fetal bovine serum (FBS, Gibco; Thermo Fisher Scientific, Waltham, MA), 100 units/mL of penicillin, and 100 μg/mL of streptomycin at 37℃ in a humidified atmosphere of 5% CO2.SUN-398 cell was cultured in Roswell Park Memorial Institute 1640 (RPMI-1640) supplemented with 10% FBS.

**Hepatocyte isolation:**

Primary hepatocytes were isolated by a two-step perfusion technique. Mice hepatocytes were isolated from the digested liver by centrifugation over Opti-Prep density gradients. Cell viability assessed by trypan blue exclusion was more than 90%. Primary hepatocytes were cultured in DMEM/10% FBS containing penicillin and streptomycin. The purity of isolated hepatocytes was demonstrated by immunofluorescence using albumin antibody.

**The construction of the plasmid expressing β2 spectrin and cell transfection**

Human β2SP(NM_178313.2) was cloned by polymerase chain reaction from the reverse transcribed cDNAs of HepG2 cell RNA. Full length human β2SP cDNA were amplified using two pairs of PCR primes, and the PCR products were cleaned and cloned respectively into pMD™19-T Vector (Takara,6013, China). The two fragments of β2SP cDNA were purified and cloned into pcDNA3.1(-) vector backbone. The sequences of the primers were as following: pair 1: forward primer; 5-**GCC ACC***ATG* GAA TTG CAG AGG ACG TCT AG -3 reverse primer; 5- CC ACA TCT TGT GGA GCT CGTT -3; **GCC ACC** is Kozak sequence. Pair 2: forward primer; 5 -CGC AGC ACG AGA ACA TCA AG -3 reverse primer; 5 -TCA ***CTT GTC ATC GTC GTC CTT GTA GTC*** CAG TCC AGA CCA TGG CTG GTC- 3; ***CTT GTC ATC GTC GTC CTT GTA GTC*** is the sequence of Flag-Tag. HCC cells were transfected with the vector expressing β2SP using Lipofectamine^TM^ 2000 (Invitrogen, Carlsbad, CA).

**RNA extraction and qRT-PCR**

Total RNA was extracted from liver tissues and the cells using Trizol reagent (Invitrogen), and 1 mg of total RNA was used for cDNA synthesis, using an RT-PCR kit (Takara, DRR037S, Dalian,China).Real-time polymerase chain reaction (PCR) was performed using Taraka SYBR® Premix Ex Taq™ (Takara, RR036A, China). Primer sequences were listed in supplementary Table 2.

**Western blot**

Lysates from cells and tissues were collected using RIPA buffer (Sigma R0278, St Louis, MO, USA). Equal amounts of protein were separated on SDS-polyacrylamide gels, immunoblotted with primary antibodies, then with horseradish peroxidase-conjugated secondary antibodies. The blot was washed three times and was developed with ECL according to the manufacturer’s instructions. Antibodies used were listed in Supplementary Table 1.

**Cell proliferation assay**

Cell proliferation was assessed by Cell Counting Kit-8 (CCK-8). CCK-8 was performed according to the manufacturer’s protocol. In brief, cells seed in a 96-well plate were treated with test reagents and the controls.CCK-8(C0038, Beyotime, China) was incorporated into proliferating cells for 2 hours. The absorbance was measured at 450 nm using a micro plate reader.

**Cell cycle assay**

Cell cycle assay using flow cytometry were done as previously described .

**Flow cytometry analysis**

To determine the proportion of EpCAM^+^ or CD133^+^ cells, the cells were incubated with phycoerythrin (PE)-conjugated anti-human CD133 antibody (Miltenyi Biotec, Bergisch Gladbach Germany) or PE-conjugated anti-human EpCAM antibody (BioLegend, San Diego,CA)on ice for 20 minutes. PE-conjugated isotype IgGs were used as controls. The data were analyzed with a FACS Calibur flow cytometer and Cell Quest software (BD Biosciences, CA).

**Isolation of CD133^+^ or EpCAM^+^ Populations from HCC cells by Magnetic Bead Cell Sorting**

Isolation of CD133^+^ or EpCAM^+^ populations from HCC cells was performed using Miltenyi MicroBead Kit. In brief, cells were incubated with FcR blocking reagent, and then labeled with CD133 microBeads (Miltenyi Biotec,130-100-857) or

EpCAM microBeads (Miltenyi Biotec,130-061-101). The separation was conducted on MACS LS column and MACS Separator (Miltenyi Biotec). All the procedures were carried out according to manufacturer’s instructions. The purity of sorted cells was analyzed with a FACS Calibur machine and CellQuest software (BD Biosciences).

**Colony formation assay**

Sorted cells were seeded in6-well plates. After cultured for 7 days, colonies consisting of more than 50 cells were counted and stained using crystal violet (Sigma-Aldrich), photographed by inverted phase contrast microscope (Olympus, Japan)and counted.

**Sphere formation assay**

To investigate the ability to form cell spheres, sorted cells were seeded in 6-well ultra-low attachment plates (Corning, Corning, NY). The cells were cultured in serum-free medium, supplemented with 20 ng/mL human recombinant EGF (Peprotech, AF-100-15; Rocky Hill, NJ), 10 ng/mL human recombinant basic fibroblast growth factor (bFGF, Peprotech, 100-18B, Rocky Hill, NJ), 4 mg/mL insulin (Sigma), 2% B27supplement (Invitrogen), 1% Methyl cellulose (Sigma-Aldrich),500 units/mL penicillin (Invitrogen), and 500 mg/mL streptomycin (Invitrogen). After 5-7 days of culturation, spheres were counted and photographed under inverted phase contrast microscope (Olympus, Japan).

**Chemotherapeutic resistance assay**

Sorted cells were seed in 96-well plates, and were treated with doxorubicin (DOX, KeyGEN, China; KGA8181) or 5-fluorouracil (5-FU, KeyGEN, China; KGA8201). Cell viability was determined by CCK8 cell counting kit and detected by Microplate Spectrophotometry at wave of 450 nm.

**Cell invasion assays**

The invasion assays were performed in the 24-well Chambers (Corning) coated with Matrigel (BD Biosciences) according to the manufacturer’s instructions. The cells were seeded with serum-free DMEM or RPMI1640 medium in the upper chamber, and10% FBS in DMEM or RPMI1640 was added to the bottom chamber as an attractant. After 48 hours of incubation, the cells that invaded through the Matrigel were stained with Crystal violet, then were counted and photographed (Olympus, Japan).

***In vivo* tumorigenicity assay**

Male 2-4 weeks NOD-SCID mice were purchased from Beijing Huafukang Company. The sorted cells were resuspended in sterile DMEM or RPMI 1640 medium without FBS, then mixed with Matrigel (BD Biosciences, CA) (1:1). 200μl mixture containing 5000 to 2500,00 cells was injected subcutaneously at opposite site in the flanks of NOD/SCID mice. The size and incidence of subcutaneous tumors were recorded. For histologic evaluation, tumors were formalin-fixed, paraffin-embedded or embedded directly in OCT compound. All procedures were incompliance with our institution’s guidelines for the use of laboratory animals and were approved by the Institution Animal Care and Use Committee.

**Xenograft tumor model and treatment**

To establish xenografts, CD133^+^ Huh7(30×10^4^ cells) or EpCAM^+^ SNU-398 cells (30×10^4^ cells) resuspended 1:1 in 200 µl of DMEM and Matrigel (BD Biosciences) and subcutaneously injected into four-week-old NOD/SCID mice. When the tumor volume reached ∼50 mm^3^ (diameter 4-5 mm), liposome-DNA complexes [DNA (25 μg)/liposome (75μl) = 1:3] were injected into the tumor or around the tumor every 3 days in a volume of 100 μL for a total of five times.TheNOD/SCID mice were randomly divided into 2 groups (n=5 each).Tumors were measured every 4 days. The tumor volume was calculated as length × width^2^/2.

Co-immunoprecipitation(Co-IP):

Lysates from cultured cells were collected using RIPA buffer, and then lysates were immunoprecipitated with primary antibody and protein A/G PLUS-Agarose (Santa Cruz, sc-2003). After washing the pellet 3-5 times, the samples received immunoblot analysis.

**Statistical analysis**

All data were expressed as mean ± standard error from 3 separate experiments performed in triplicate except otherwise noted. The differences between groups were analyzed by Student’s t test and *P* < 0.05 was considered to be statistically significant.

Supplementary Table 1. Primary antibodies used in our study.

| **Antigens** | **species** | **Manufacturer** | **Application** | **Catalogue number** |
| --- | --- | --- | --- | --- |
| HNF4α | rabbit | Abcam | IHC | ab201460 |
| β-catenin | rabbit | Proteintech | Immunofluorescence | 51067-2-AP |
| SMAD3 | rabbit | Proteintech | Immunofluorescence | 25494-1-AP |
| SMAD4 | rabbit | Proteintech | Immunofluorescence | 51069-2-AP |
| βspectrin | rabbit | Abcam | IHC  Western  Co-IP BlottingImmunofluorescence | ab72239 |
| ALB | rabbit | Proteintech | IHC | 16475-1-AP |
| Cyclin A2 | rabbit | Epitomics | Western Blotting | 1547-1 |
| Cyclin B1 | rabbit | Epitomics | Western Blotting | 1495-1 |
| Cyclin D1 | rabbit | Epitomics | Western Blotting | 2261-1 |
| Cyclin E1 | rabbit | Epitomics | Western Blotting | 3327-1 |
| CDK4 | rabbit | Epitomics | Western Blotting | 3830-1 |
| p53 | rabbit | Epitomics | Western Blotting | 1026-1 |
| pRb | rabbit | Epitomics | Western Blotting | 3408-1 |
| β-actin | rabbit | Abcam | Western Blotting | ab8227 |
| CD133 | mouse | MiltenyiBiotech | Flow cytometry | 130-098-826 |
| EpCAM | mouse | BioLegend Inc | Flow cytometry | 324205 |
| CD90 | mouse | Miltenyi Biotech | Flow cytometry | 130-097-932 |
| CK19 | rabbit | Proteintech | IHC | 14965-1-AP |
| CD133 | rabbit | Proteintech | IHC | 1840-1-AP |
| EpCAM | rabbit | Abcam | IHC | ab71916 |
| CD 90 | rabbit | Abcam | Western Blotting | Ab133350 |
| CK19 | rabbit | Proteintech | Western Blotting | 10712-1-AP |
| HNF1α | rabbit | Proteintech | Western Blotting | 22426-1-AP |
| AFP | rabbit | Proteintech | Western Blotting | 14550-1-AP |
| EpCAM | rabbit | Proteintech | Western Blotting | 21050-1-AP |
| (Total) β-Catenin | rabbit | CST | Western Blotting  Co-IP | 8480 |

| **Supplementary table 2: Primer sequences of RT-PCR used in our study** | | |
| --- | --- | --- |
| **Gene** | **Forward primer(5′- 3′)** | **Reverse primer(5′- 3′)** |
| **Liver-specific genes** | | |
| ***Human*** | | |
| ALB(NM_000477) | TGCACAGAATCCTTGGTGAA | TTCACGAGCTCAACAAGTGC |
| HNF1α(NM_000545) | CCATCCTCAAAGAGCTGGAG | TGTTGTGCTGCTGCAGGTA |
| HNF4α(NM_000457) | CTGCACCCTCACCTGATGC | GGCTGGGGGATGGCAGAG |
| OAT(NM_000274) | TATCTCCAGTTCCACAGACC | TCACCCTGAATTGGTTCTAC |
| CRP(NM_000567) | CAGACAGACATGTCGAGGAAGG | AGGCTTTGAGAGGCTTCGTT |
| G6PC(NM_000151) | CGTGCCCCTGATAAAGCAGT | GTATACACCTGCTGTGCCCAT |
| ADH1(NM_000667) | GGCTCTACCTGTGCTGTGTT | CCCAACTCTTTGGCCTTTGC |
| CYP7A1(NM_000780) | CAGAACTGAATGACCTGCCA | GGTGCAAAGTGAAATCCTCC |
| ***Rat*** | | |
| AFP(NM_012493) | CGCAAAAGAGGGTCCAAAGT | ACATGGAAGTCTCCACCAGT |
| Albumin(NM_134326) | TTCAAAGGCCTAGTCCTGATTG | TGTCACAGTTTTCGGCATTCT |
| G6PC(NM_013098) | TCTTGGTGTCTGTGATCGCT | CGCTGTCCAAAAAGAATCCACT |
| HNF4(NM_001270931) | CACATGGGCACCAATGTCAT | TCTGTCCATTGCTGAGGTGAGA |
| HNF1(NM012669) | GTCCCACAGTGTCCTCCAG | CCCTCCTCTCCCCCTGT |
| **Liver cancer stem cell markers** | | |
| ***Human*** | | |
| CD90(NM_006288) | CGCTCTCCTGCTAACAGTCTT | CGTGCTTCTTTGTCTCACGG |
| CD133(NM_001145847) | ATCTGCAGTGGATCGAGTTCTCT | GCGGTGGCCACAGGTTT |
| EpCAM(NM_002354) | GCTGGCCGTAAACTGCTTTG | ACATTTGGCAGCCAGCTTTG |
| CK19(NM_002276) | GAGCATGAAAGCTGCCTTGG | GTACTCCTGATTCTGCCGCT |
| **Others** |  |  |
| Human β2SP (NM_178313.2） | GGAAATCCCTGTTGGCGAGA | CTGGCTGTCCGTCTGCTATT |
| Human GAPDH(NM_001256799) | GAGAAGGCTGGGGCTCATTT | GTCAAAGGTGGAGGAGTGGG |
| Human β-catenin (NM-001330729.1） | AAGGCTACTGTTGGATTGATTCG | ACGCAAAGGTGCATGATTTG |
| Rat β2SP (NM_001013130) | CGAAGTGGCCCTTGATTACAA | GAGGTACTCGTTGCCATCACTTAG |
| Rat β actin(NM_031144) | TGACAGGATGCAGAAGGAGAT | GATAGAGCCACCAATCCACACA |

**References:**

1. Schiffer E*, et al.* Gefitinib, an EGFR inhibitor, prevents hepatocellular carcinoma development in the rat liver with cirrhosis. *Hepatology* 2005, **41**(2)**:** 307-314.

2. Newell P*, et al.* Experimental models of hepatocellular carcinoma. *Journal of hepatology* 2008, **48**(5)**:** 858-879.

3. Sun W*, et al.* Gankyrin-mediated dedifferentiation facilitates the tumorigenicity of rat hepatocytes and hepatoma cells. *Hepatology* 2011, **54**(4)**:** 1259-1272.

4. Wang Z*, et al.* Embryonic liver fodrin involved in hepatic stellate cell activation and formation of regenerative nodule in liver cirrhosis. *Journal of cellular and molecular medicine* 2012, **16**(1)**:** 118-128.

5. Wang Z*, et al.* beta-2 spectrin is involved in hepatocyte proliferation through the interaction of TGFbeta/Smad and PI3K/AKT signalling. *Liver international : official journal of the International Association for the Study of the Liver* 2012, **32**(7)**:** 1103-1111.

6. He X*, et al.* CP-31398 prevents the growth of p53-mutated colorectal cancer cells in vitro and in vivo. *Tumour biology : the journal of the International Society for Oncodevelopmental Biology and Medicine* 2015, **36**(3)**:** 1437-1444.

7. He X*, et al.* Functional repair of p53 mutation in colorectal cancer cells using trans-splicing. *Oncotarget* 2015, **6**(4)**:** 2034-2045.

8. He X*, et al.* Trans-splicing repair of mutant p53 suppresses the growth of hepatocellular carcinoma cells in vitro and in vivo. *Scientific reports* 2015, **5:** 8705.

9. He XX*, et al.* CP-31398 inhibits the growth of p53-mutated liver cancer cells in vitro and in vivo. *Tumour biology : the journal of the International Society for Oncodevelopmental Biology and Medicine* 2016, **37**(1)**:** 807-815.
